# Integrative taxonomy of the genus *Dudgeodes* Sartori, 2008 (Insecta, Ephemeroptera, Teloganodidae) from the Philippines with description of new species and supplementary descriptions of Southeast Asian species

**DOI:** 10.3897/zookeys.910.48659

**Published:** 2020-02-10

**Authors:** Jhoana M. Garces, Michel Sartori, Hendrik Freitag

**Affiliations:** 1 Department of Biology, School of Science and Engineering, Ateneo de Manila University, Quezon City, Philippines Ateneo de Manila University Quezon City Philippines; 2 Museum of Zoology, Palais de Rumine, Place Riponne 6, CH-1014 Lausanne, Switzerland Museum of Zoology Lausanne Switzerland; 3 Department of Ecology and Evolution, Biophore, University of Lausanne, CH-1015 Lausanne, Switzerland University of Lausanne Lausanne Switzerland

**Keywords:** Ephemerelloidea, Luzon, Mindoro, Negros, Oriental Region, Pannota

## Abstract

COI sequences were used as an initial clustering method to delimit putative species of the genus *Dudgeodes* in the Philippines. An overview of the diagnostic characters of Philippine species and characters with high intraspecific variability are given. Six new species of *Dudgeodes* are described and illustrated: *D. bauernfeindi* Garces & Sartori, **sp. nov.**, *D. freitagi* Garces & Sartori, **sp. nov.**, *D. luntian* Garces & Sartori, **sp. nov.**, *D. pangantihoni* Garces & Sartori, **sp. nov.**, *D. tabang* Garces & Sartori, **sp. nov.**, and *D. vonrinteleni* Garces & Sartori, **sp. nov.**, all known from the nymphal stage. Supplementary descriptions are provided for *D. pescadori* Sartori, 2008, *D. hutanis* Sartori, 2008, *D. stephani* Sartori, 2008, *D. ulmeri* Sartori, 2008, and *D. celebensis* Sartori, 2008. A key to the nymphs of Philippine *Dudgeodes* species is proposed.

## Introduction

The use of short DNA fragment sequences, such as the cytochrome oxidase subunit 1 (COI), is recommended as an efficient start of the taxonomic pipeline to cluster putative new species for further assessment (Monaghan et al. 2005, Balke et al. 2013, Kekkonen and Hebert 2014, deWaard et al. 2019). Incorporating this process in the taxonomic pipeline has been shown to improve the rate of species discovery especially on hyperdiverse taxa (Kaltenbach and Gattolliat 2018, Meierotto et al. 2019, Riedel and Narakusumo 2019). To a certain extent, “turbo-taxonomy” is proposed which involves initial clustering of samples using COI, followed by concise morphological descriptions and high-resolution digital imaging (Riedel et al. 2013).

Aside from usefulness in large-scale biodiversity inventory, incorporating DNA-based clustering method is also useful in improving taxonomic work efficiency (Riedel et al. 2013, Kekkonen and Hebert 2014). This is particularly useful for aquatic insects where species descriptions may be based on nymphs with various morphological characters displaying diverse intraspecific differences. Elucidating nymph-based taxonomy is arguably favored in taxa like mayflies (Ephemeroptera) where there is high motivation to study the nymphs owing to biomonitoring programs (Boonsoong et al. 2008).

The small mayfly family Teloganodidae (Allen 1965) is recorded from Afrotropical to Oriental realms and encompasses 9 genera and 25 species (Barber-James et al. 2008, Sartori et al. 2008, Selvakumar et al. 2014, Anbalagan et al. 2015, Martynov et al. 2016). The most speciose genus, *Dudgeodes* Sartori, 2008 currently has nine species: *D. bharathidasani* Anbalagan, 2015 (India), *D. celebensis* Sartori, 2008 (Sulawesi), *D. hutanis* Sartori, 2008 (Borneo), *D. lugens* (Navás, 1933) (China), *D. palnius* Selvakumar, Sivamarakrishnan & Jacobus, 2014 (India), *D. pescadori* Sartori, 2008 (Philippines), *D. romani* Martynov, Palatov & Boonsoong, 2016 (Thailand), *D. stephani* Sartori, 2008 (Borneo), and *D. ulmeri* Sartori, 2008 (Java, Sumatra).

Despite the notable diversity of the Philippine archipelago, only *Dudgeodes pescadori* from Luzon island has been reported. Given the current distribution and ecological information (Martynov et al. 2016), there is a high probability that the genus is present across the entire country. Most biomonitoring studies in the country used identification keys that are not suitable for the Philippine archipelago; hence it is likely that Teloganodidae and genus *Dudgeodes* were reported under the family name Ephemerellidae before (Flores and Zafaralla 2012).

In this contribution we use COI sequences as an initial clustering method to delimit putative species of the genus *Dudgeodes* in the Philippines. For clusters of more than five individuals, we proceeded with morphological analysis to identify characters that were most useful in Philippine species identification and characters with high intraspecific variability. Consequently, six new species of *Dudgeodes* from the Philippines are described herein. In addition, supplementary descriptions of some Southeast Asia *Dudgeodes* species are also provided.

## Materials and methods

The specimens used in the study were mainly collected by the members the Biodiversity Laboratory, Ateneo de Manila University (Philippines). Vouchered specimen from the type locality of *Dudgeodes pescadori* and conspecific *D. romani* from Cambodia (additional material of the field report of Freitag et al. (2018)) were also included in the study for comparison. Specimens were preserved in > 96% ethanol. Each *Dudgeodes* nymph specimen was assigned an EPH ### code for subsequent analysis.

DNA was extracted from one set of legs of each EPH sample by elution with Qiagen DNeasy kit (Qiagen, Hilden, Germany) following the protocol for animal tissues. For samples with successful DNA isolations, amplification of the cytochrome c oxidase subunit 1 (COI) was performed following the procedures and primers described in Garces et al. (2018). Successful amplifications were sent to Macrogen Europe B.V. (Netherlands) for cleaning and cycle sequencing.

Forward and reverse sequences were assembled and edited using BioEdit 7.0.5.3 (Hall 1999). Multiple sequence alignment and checking for stop codons and indels were done in MEGA X (Kumar et al. 2018). Nucleotide sequences obtained in this study were deposited in GenBank (Accession Numbers MN853779–MN853855) as listed in Table 1.

**Table 1.** COI GenBank accession numbers, voucher information and GenSeq nomenclature of *Dudgeodes* species included in this study.

Species	Specimen code	Locality GPS codes	Sex	GenBank	GenSeq nomenclature
*D. bauernfeindi* sp. nov.	EPH 150	09°18'22"N, 123°10'04"E	Male	MN853779	genseq-2 COI
	EPH 155	09°18'N, 123°14'E	Female	MN853780	genseq-2 COI
	EPH 157	09°17'N, 123°13'E	Male	MN853781	genseq-2 COI
	EPH 158	09°17'N, 123°13'E	Female	MN853782	genseq-2 COI
	EPH 160	09°17'N, 123°13'E	Male	MN853783	genseq-2 COI
	EPH 356	09°18'N, 123°14'E	Male	MN853784	genseq-1 COI
	EPH 357	09°18'N, 123°14'E	Male	MN853785	genseq-2 COI
*D. freitagi* sp. nov.	EPH 146	13°40'43"N, 121°15'04"E	Female	MN853786	genseq-2 COI
	EPH 147	13°40'43"N, 121°15'04"E	Female	MN853787	genseq-2 COI
	EPH 148	13°40'43"N, 121°15'04"E	Male	MN853788	genseq-2 COI
	EPH 184	14°08'N, 121°31'E	Male	MN853789	genseq-2 COI
	EPH 191	14°08'N, 121°31'E	Female	MN853790	genseq-1 COI
	EPH 238	15°06'N, 121°06'E	Female	MN853791	genseq-2 COI
	EPH 245	14°48'59"N, 120°20'31"E	Male	MN853792	genseq-2 COI
	EPH 246	14°48'59"N, 120°20'31"E	Female	MN853793	genseq-2 COI
*D. luntian* sp. nov.	EPH 127	12°37.30'N, 121°22.97'E	Male	MN853794	genseq-2 COI
	EPH 136	14°24'55"N, 121°29'36"E	Male	MN853795	genseq-2 COI
	EPH 137	14°24'55"N, 121°29'36"E	Male	MN853796	genseq-1 COI
	EPH 149	09°18'17"N, 123°10'07"E	Male	MN853797	genseq-2 COI
	EPH 151	09°18'22"N, 123°10'04"E	Male	MN853798	genseq-2 COI
	EPH 152	09°18'22"N, 123°10'04"E	Male	MN853799	genseq-2 COI
	EPH 153	09°18'N, 123°14'E	Male	MN853800	genseq-2 COI
	EPH 154	09°18'N, 123°14'E	Male	MN853801	genseq-2 COI
	EPH 156	09°18'N, 123°14'E	Male	MN853802	genseq-2 COI
	EPH 159	09°18'N, 123°14'E	Female	MN853803	genseq-2 COI
	EPH 173	13°52'28"N, 122°56'38"E	Female	MN853804	genseq-2 COI
	EPH 185	14°08'N, 121°31'E	Female	MN853805	genseq-2 COI
	EPH 186	14°08'N, 121°31'E	Male	MN853806	genseq-2 COI
	EPH 239	09°18'22"N, 123°10'04"E	Female	MN853807	genseq-2 COI
	EPH 247	14°46'22"N, 120°17'50"E	Female	MN853808	genseq-2 COI
*D. pangantihoni* sp. nov.	EPH 163	09°06'39"N, 124°43'45"E	Female	MN853809	genseq-2 COI
	EPH 165	8°28'N, 125°59'E	Male	MN853810	genseq-2 COI
	EPH 166	09°06'39"N, 124°43'45"E	Male	MN853811	genseq-2 COI
	EPH 168	09°06'39"N, 124°43'45"E	Male	MN853812	genseq-2 COI
	EPH 169	8°28'N, 125°59'E	Female	MN853813	genseq-2 COI
	EPH 207	8°15'10"N, 124°34'51"E	Male	MN853814	genseq-2 COI
	EPH 208	8°15'10"N, 124°34'51"E	Female	MN853815	genseq-2 COI
	EPH 209	9°11'34"N, 125°36'34"E	Male	MN853816	genseq-2 COI
	EPH 210	9°11'34"N, 125°36'34"E	Male	MN853817	genseq-2 COI
	EPH 211	9°11'34"N, 125°36'34"E	Female	MN853818	genseq-2 COI
	EPH 212	8°15'10"N, 125°02'07"E	Male	MN853819	genseq-2 COI
	EPH 213	8°15'07"N, 124°34'56"E	Male	MN853820	genseq-2 COI
	EPH 220	09°06'39"N, 124°43'45"E	Male	MN853821	genseq-1 COI
	EPH 221	09°06'39"N, 124°43'45"E	Female	MN853822	genseq-2 COI
*D. pescadori* Sartori, 2008	EPH 130	14°09'54"N, 121°14'50"E	Male	MN853823	genseq-4 COI
	EPH 135	14°09'54"N, 121°14'50"E	Female	MN853824	genseq-4 COI
	EPH 248	14°50'32"N, 120°21'30"E	Female	MN853825	genseq-4 COI
*D. romani* Martynov, Palatov & Boonsoong, 2016	EPH 138	11°21'58"N, 104°06'17"E	Female	MN853826	genseq-4 COI
	EPH 139	11°23'00"N, 104°06'50"E	Male	MN853827	genseq-4 COI
*D. tabang* sp. nov.	EPH 193	11°04'37"N, 124°41'46"E	Male	MN853828	genseq-2 COI
	EPH 194	11°04'37"N, 124°41'46"E	Female	MN853829	genseq-1 COI
	EPH 331	11°03'55"N, 124°42'17"E	Male	MN853830	genseq-2 COI
	EPH 332	11°03'55"N, 124°42'17"E	Male	MN853831	genseq-2 COI
	EPH 333	11°03'55"N, 124°42'17"E	Male	MN853832	genseq-2 COI
	EPH 334	11°03'55"N, 124°42'17"E	Male	MN853833	genseq-2 COI
	EPH 335	11°03'55"N, 124°42'17"E	Female	MN853834	genseq-2 COI
*D. vonrinteleni* sp. nov.	EPH 180	14°06'60"N, 121°27'54"E	Female	MN853835	genseq-2 COI
	EPH 181	14°06'60"N, 121°27'54"E	Female	MN853836	genseq-2 COI
	EPH 182	14°06'60"N, 121°27'54"E	Female	MN853837	genseq-2 COI
	EPH 188	14°06'42"N, 121°30'19"E	Female	MN853838	genseq-2 COI
	EPH 189	14°06'42"N, 121°30'19"E	Male	MN853839	genseq-2 COI
	EPH 342	14°06'60"N, 121°27'54"E	Male	MN853840	genseq-2 COI
	EPH 343	14°06'60"N, 121°27'54"E	Female	MN853841	genseq-2 COI
	EPH 344	14°06'60"N, 121°27'54"E	Female	MN853842	genseq-2 COI
	EPH 345	14°06'60"N, 121°27'54"E	Female	MN853843	genseq-1 COI
*Dudgeodes* PH1	EPH 192	11°04'37"N, 124°41'46"E	Male	MN853844	genseq-4 COI
*Dudgeodes* PH2	EPH 198	10°41'10"N, 123°10'43"E	Male	MN853845	genseq-4 COI
	EPH 199	10°41'10"N, 123°10'43"E	Male	MN853846	genseq-4 COI
*Dudgeodes* PH3	EPH 243	11°37'11"N, 125°23'37"E	Male	MN853847	genseq-4 COI
*Dudgeodes* PH4	EPH 241	8°56'N, 117°50'E	Male	MN853848	genseq-4 COI
*Dudgeodes* PH5	EPH 187	14°08'N, 121°31'E	Female	MN853849	genseq-4 COI
	EPH 337	14°06'42"N, 121°30'19"E	Male	MN853850	genseq-4 COI
	EPH 190	14°06'42"N, 121°30'19"E	Male	MN853851	genseq-4 COI
	EPH 183	14°06'60"N, 121°27'54"E	Male	MN853852	genseq-4 COI
*Dudgeodes* PH6	EPH 252	10°00'43"N, 119°01'07"E	Female	MN853853	genseq-4 COI
	EPH 253	10°28'10"N, 119°19'52"E	Female	MN853854	genseq-4 COI
*Dudgeodes* PH7	EPH 214	8°15'07"N, 124°34'56"E	Female	MN853855	genseq-4 COI

Different species delimitation methods were used to generate molecular species clusters. First, general mixed Yule coalescence (GMYC) (Pons et al. 2006, Fujisawa and Barraclough 2013) was done using ultrametric gene tree as input reconstructed using methods described in Rutschmann et al. (2014) in BEAST v1.10.4 (Suchard et al. 2018). Two inferences were run, and all parameters reached effective sample size > 400. Single threshold GMYC analysis was conducted using the Splits package (Ezard et al. 2018) for R v3.5.2 (R Development Core Team 2017). Second species delimitation method is Poisson Tree Processes (PTP) (Zhang et al. 2013) using Maximum Likelihood (ML) tree inferred from MEGA X following best model Tamura-3 parameter+GI from AICc. *Teloganodes kodai* was chosen as the outgroup (Selvakumar et al. 2016). Single PTP method (p value 0.001) and multiple PTP (mPTP) (Kapli et al. 2017) with default settings available from the webserver was used. To illustrate different haplogroups with respect to intra-Philippine biogeographic region, statistical parsimony network was done in PopART v1.7 (Leigh and Bryant 2015).

**Figure 1.**
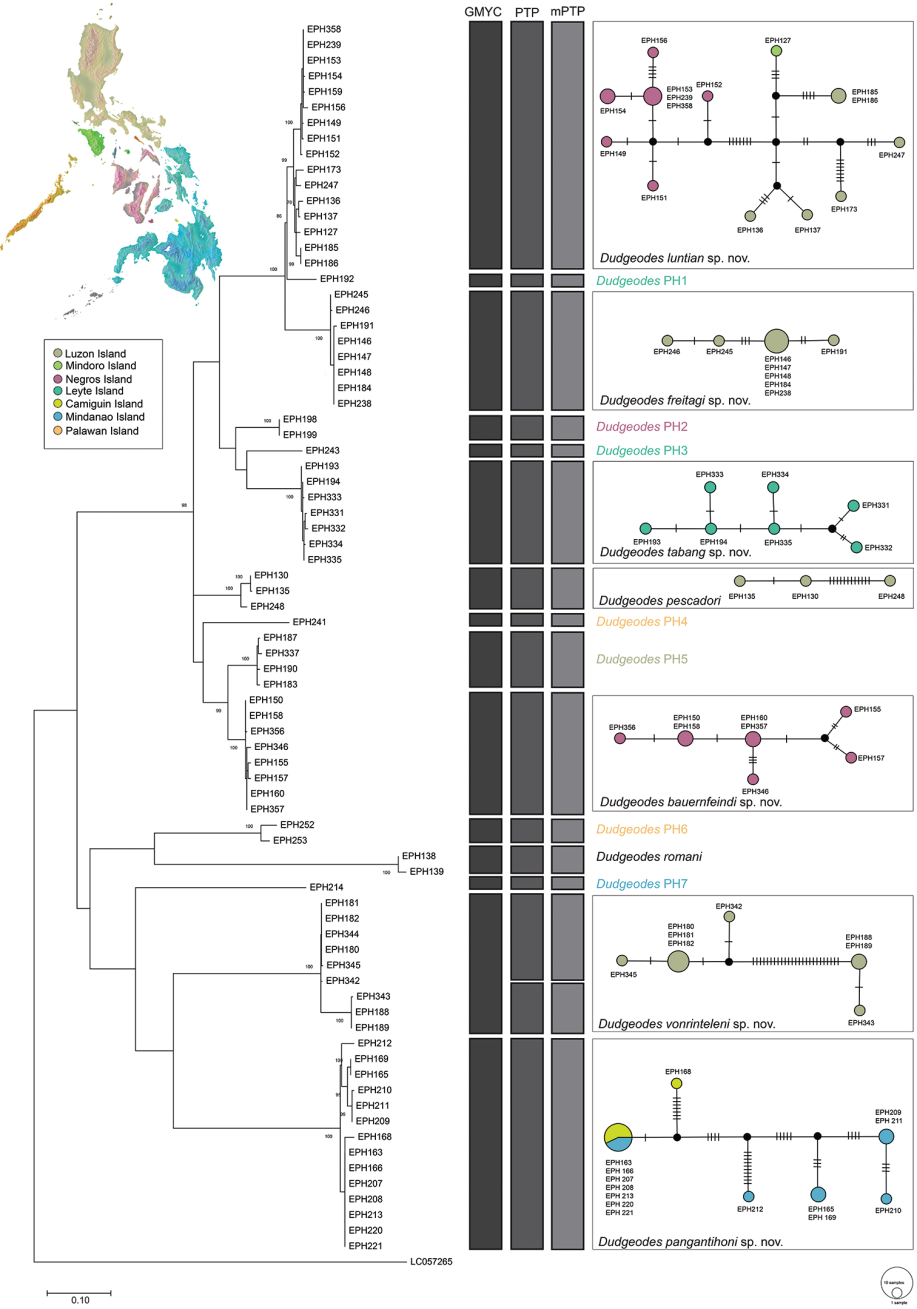
Molecular species delimitation of Southeast Asian *Dudgeodes* using generalized mixed Yule coalescence (GMYC), Poisson Tree Processes (PTP) and multi-rate Poisson Tree Processes (mPTP) using partial COI sequence (593 bp). The phylogenetic tree shows the topology of COI gene tree following Maximum Likelihood method and Tamura 3-parameter+GI model, 1000 bootstrap. Only nodes with bootstrap > 70% are indicated. Statistical parsimony network of the new species and *D. pescadori* are given depicting the haplotype diversity and localities. Colors based on intra-Philippine biogeographic regions; black circles correspond to intermediate or missing haplotypes.

Molecular species hypotheses generated by the different methods were summarized in Fig. 1 and served as the initial clustering. Morphological analysis was conducted in all clusters with more than five individuals to verify molecular operational units and were described in detail to allow for observation of intraspecific variability. Clusters with fewer than five individuals were given provisional species designations and will be treated in detail in the future when more material is available.

Voucher specimen were photographed using Canon EOS 6D camera with the Visionary Digital LK imaging system (Dun Inc., Virginia) and processed with the programs Adobe Photoshop Lightroom and Helicon Focus version 5.3. Permanent slides were made by dissecting the nymph in Cellosolve (2-Ethoxyethanol) (HiMedia Ltd. India) and mounted on slides with Euparal liquid (Carl Roth GmbH, Karlsruhe, Germany). Diagnostic characters were illustrated using Adobe Illustrator C6 following the recommendations of Coleman (2003, 2009).

Paratypes of other Southeast Asian species of *Dudgeodes* deposited in the Museum of Zoology, Lausanne, Switzerland (**MZL**) were examined for morphological comparisons and supplementary descriptions. Terminologies followed Sartori et al. (2008) and Martynov et al. (2016).

Type specimens of the species described here are deposited in the following institutions: Museum of Natural History of the Philippine National Museum, Manila, Philippines (**PNM**), Ateneo de Manila University, Quezon City, Philippines (**AdMU**), and Zoological State Collection Munich, Germany (**ZSM**), temporarily stored in MZL.

## Results

### Clustering based on COI gene data

The results of molecular species delimitation methods (Fig. 1) were very similar, ranging from 15 to 16 putative species based on the analysis of 79 COI sequences. Single threshold GMYC (p < 0.001) delineated the least number of entities consisting of eleven clusters and four singletons. The mPTP and PTP models both recovered 16 entities, including a best score coalescent rate of 210.93 and 291.21, respectively.

Based on the molecular species delimitation methods, six clusters with at least five individuals are recognized and closely examined morphologically. In addition, the cluster with specimens from the type locality of *Dudgeodes pescadori* is examined. Consequently, six new species of *Dudgeodes* are described. Supplementary descriptions of *D. pescadori*, *D. hutanis*, *D. stephani*, *D. ulmeri*, and *D. celebensis* are also given.

### Evaluation of character variability

This section summarizes characters that are similar across Philippine representatives and gives an overview of the characters which are sufficiently different that they can be used to separate the different Philippine species. Moreover, brief discussion of the advantages and disadvantages of some characters used are also included, most especially on characters which may be highly variable across different nymphal stage (i.e., with dark wings pads compared to younger nymph). Only nymphal characters are included since most imaginal stages are unknown.

General coloration

**Figure 2.**
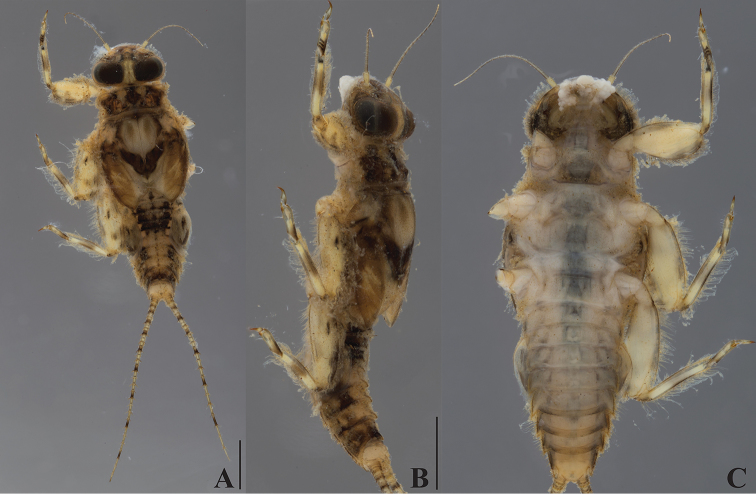
*Dudgeodes bauernfeindi* sp. nov. **A** dorsal **B** lateral **C** ventral. Scale bars: 1 mm (**B** = **C**).

**Figure 3.**
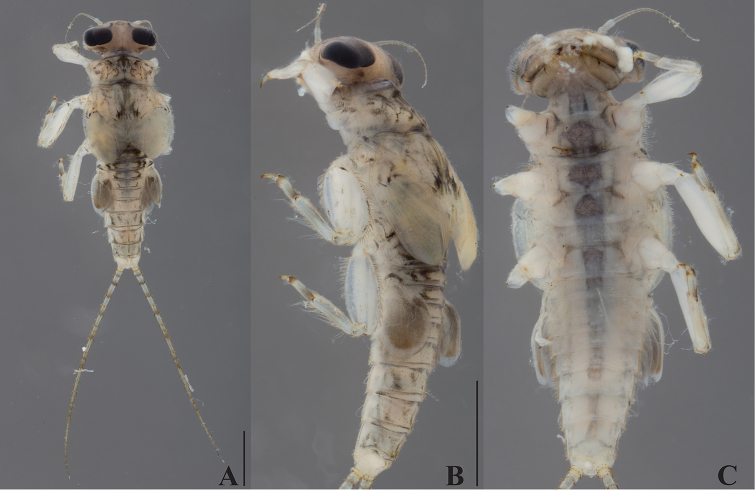
*Dudgeodes freitagi* sp. nov. **A** dorsal **B** lateral **C** ventral. Scale bars: 1 mm (**B** = **C**).

**Figure 4.**
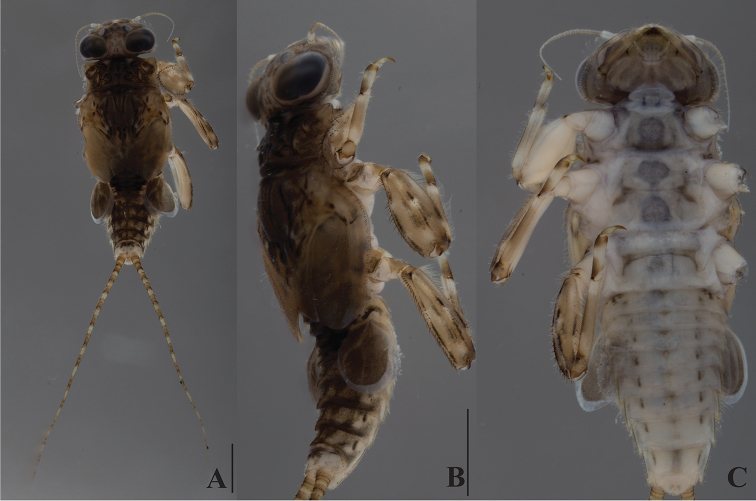
*Dudgeodes luntian* sp. nov. **A** dorsal **B** lateral **C** ventral. Scale bars: 1 mm (**B** = **C**).

**Figure 5.**
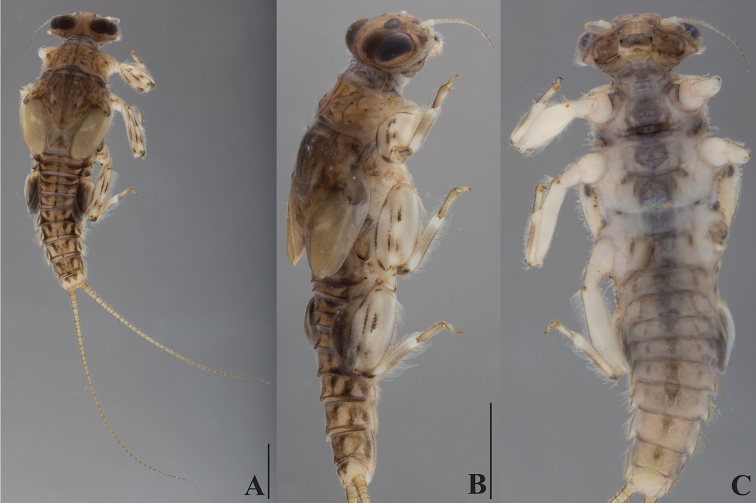
*Dudgeodes pangantihoni* sp. nov. **A** dorsal **B** lateral **C** ventral. Scale bars: 1 mm (**B** = **C**).

**Figure 6.**
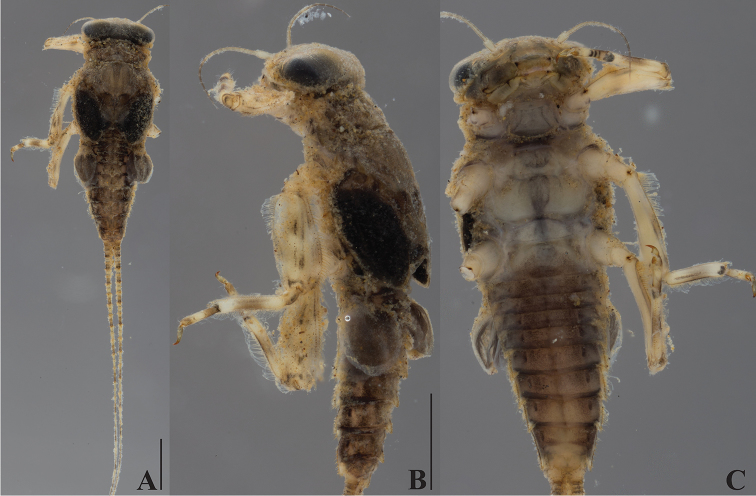
*Dudgeodes tabang* sp. nov. **A** dorsal **B** lateral **C** ventral. Scale bars: 1 mm (**B** = **C**).

**Figure 7.**
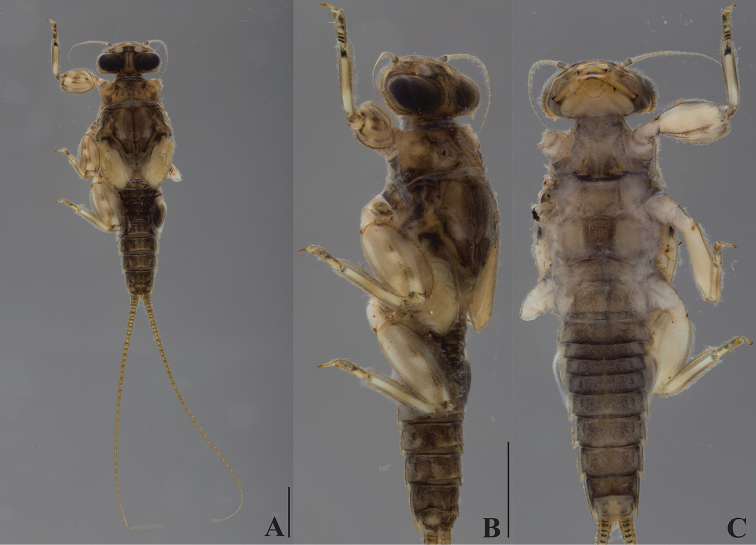
*Dudgeodes vonrinteleni* sp. nov. **A** dorsal **B** lateral **C** ventral. Scale bars: 1 mm (**B** = **C**).

**Figure 8.**
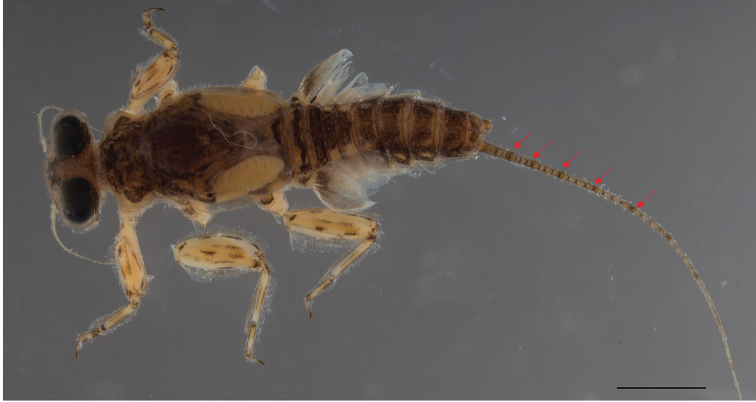
*Dudgeodes pescadori* Sartori, 2008 Paratype. Sample collected on 1977. Red arrows pointing to brownish band. Scale bar: 1 mm.

General coloration is very variable depending on the age of the nymph or preservation of the specimen, ranging from yellowish brown to dark brown (Figs 2A, 3A, 4A, 5A, 6A, 7A). Young specimens are generally paler and whitish. Head capsule and antennae are yellowish brown to brown, and the distal end of antenna may appear darker. Legs are light to middle brown, with femora bearing the four characteristic maculae (sensu Sartori et al. 2008) (Figs 2B, 3B, 4B, 5B, 6B, 7B, 8). Gills yellowish brown to dark brown but depending on the preservation, they may appear reddish.

Head

**Figure 9.**
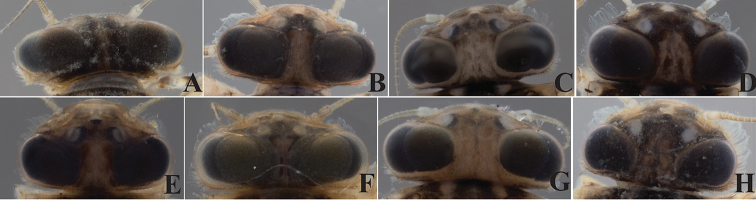
Blackish dorsal part of male eyes color across conspecific samples. *Dudgeodes luntian* sp. nov. **A** EPH127 **B** EPH136 **C** EPH137 **D** EPH149 **E** EPH151 **F** EPH153 **G** EPH156 **H** EPH186.

**Figure 10.**
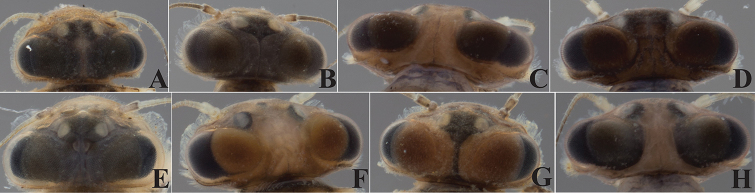
Brown dorsal part of male eyes color across conspecific samples. *Dudgeodes pangantihoni* sp. nov. **A** EPH165 **B** EPH166 **C** EPH168 **D** EPH207 **E** EPH210 **F** EPH212 **G** EPH213 **H** EPH220.

The color of the dorsal part of the male eyes may appear brown as in *Dudgeodes bauernfeindi* sp. nov., *D. pangantihoni* sp. nov., *D. tabang* sp. nov., or black as in *D. freitagi* sp. nov., *D. luntian* sp. nov., *D. vonrinteleni* sp. nov. However, the color may appear darker in older nymphs with dark wing pads already, hence brown eyes may eventually look darker in older nymphs (Figs 9A–H, 10A–H).

As reported in Sartori et al. 2008, the antenna length relative to the head width also varies between species. While most Philippine species reported here have an antenna length subequal to the head width, in *Dudgeodes luntian* sp. nov. antenna length is shorter than the head width and longer in *D. freitagi* sp. nov.

Aside from the dorsal part of male eyes and antenna length, ornamentation on the head capsule is generally less informative to differentiate the Philippine species. Occipital tubercles are absent. Head surface has few forked short thick setae and short thin setae. Like other *Dudgeodes* species, all species have outer margin of head fringed with a row of short, basally forked setae from the area in front of eyes to the labrum insertion (Martynov et al. 2016: fig. 3).

Mouthparts

Mouthparts are generally useful for generic diagnosis but less informative in differentiating Philippine species from one another.

The labrum is generally consistent in terms of its wide shape and dorsal surface with all simple setae arranged in multiple rows, like *Dudgeodes pescadori* and *D. hutanis* (Sartori et al. 2008: figs 22, 23). The degree of concavity in the central part of the anterior margin may be of diagnostic significance. However, measurements are highly affected by the slide preparation. Even for conspecific specimens, a range cannot be established. Here we report them as slightly concave as in *D. freitagi* sp. nov., *D. luntian* sp. nov., *D. pangantihoni* sp. nov., *D. vonrinteleni* sp. nov. or concave as in *D. bauernfeindi* sp. nov. and *D. tabang* sp. nov. The concave anterior margin of Philippine species is covered with numerous feathered setae.

**Figure 11.**
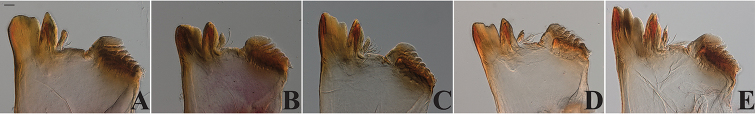
Outer incisor variability across conspecific samples. *Dudgeodes pangantihoni* sp. nov. **A** EPH163 **B** EPH165 **C** EPH168 **D** EPH207 **E** EPH212. Scale bar: 20 μm.

**Figure 12.**
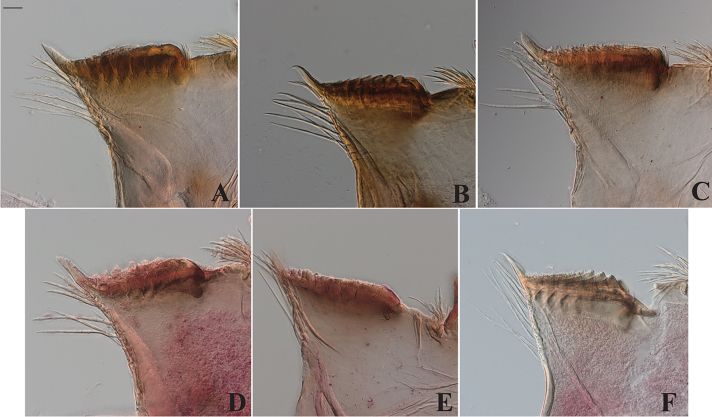
Row of simple and feathery long setae below the right mandible mola of *Dudgeodes* species. **A***D. pangantihoni* sp. nov. **B***D. luntian* sp. nov. **C***D. vonrinteleni* sp. nov. **D***D. bauernfeindi* sp. nov. **E***D. tabang* sp. nov. **F***D. freitagi* sp. nov. Scale bar: 20 μm (**A–E**).

Mandibles are slender with one long thin seta in the middle of the outer margin, and few shorter setae in the proximal half of the margin. Both outer incisors are composed of three teeth and the inner incisors with two teeth. As noted in Sartori et al. (2008), mandibles are difficult to interpret because they are subject to wear and tear. The shape of the outer incisor highly varies between different nymphal stages (Fig. 11A–E). Prostheca are reduced with a group of setae. The row of long setae below the right mandible mola appears to be a combination of simple and feathery (Fig. 12A–F), in contrast to the simple hair-like setae in *Dudgeodes romani*.

Maxillary palp is reduced into a small knob with a single simple seta (Martynov et al. 2016: fig. 10). The inner margin at the base of lacinia has one long (ventral) and four or five shorter pointed setae with feathered margin (Sartori et al. 2008: fig. 44).

The hypopharynx is generally homogenous, genus typical and not informative. The general shape and ornamentation of labium is the same across Philippine species.

The labium has glossae and paraglossae short and broad, rounded apically (Sartori et al. 2008: figs 47, 49). The paraglossae are broader than glossae and the apices are densely covered with feathered setae. Labial palp is always three-segmented with articulation between segments I and II clearly visible. The segment III is generally developed and elongated. In Martynov et al. 2016, small solitary setae were noted to be present in labial palp segment III of *Dudgeodes romani*. However, it is difficult to note the presence or absence of the setae on segment III because the irregular sparse row of long simple setae on the outer margins of segments I and II are obstructing the view. The submentum is moderately developed laterally with simple setae sparsely covering the surface.

Thorax

**Figure 13.**
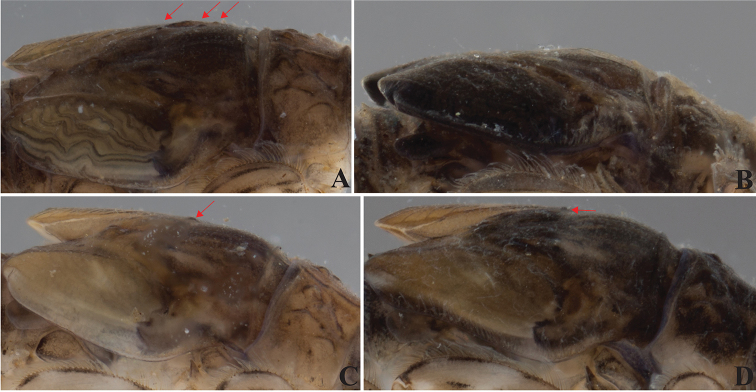
Thorax tubercles variability across nymphal stage and sex. *Dudgeodes pangantihoni* sp. nov. **A** EPH 163 female **B** EPH166 mature male **C** EPH168 male **D** EPH 207 male. Red arrows pointing mesothorax tubercles.

The pronotum and mesonotum tubercles may be significant in differentiating the Philippine species. However, the mesonotum tubercles vary depending on maturity of the nymph, with tubercles being less pronounced and difficult to discern in mature nymphs with dark wing pads (Fig. 13A–D). In contrast, the outer marginal setae of the mesonotum are more useful; they can be forked as in *Dudgeodes freitagi* sp. nov., *D. luntian* sp. nov., *D. pangantihoni* sp. nov., *D. pescadori*, *D. tabang* sp. nov., and *D. vonrinteleni* sp. nov., or simple as in *D. bauernfeindi* sp. nov. Other ornamentations are less informative. The outer pronotum margin always bears forked setae, and the pronotum and mesonotum surfaces are covered with hair-like setae.

Legs

Several characters in the legs can be used to differentiate the Philippine species.

**Figure 14.**
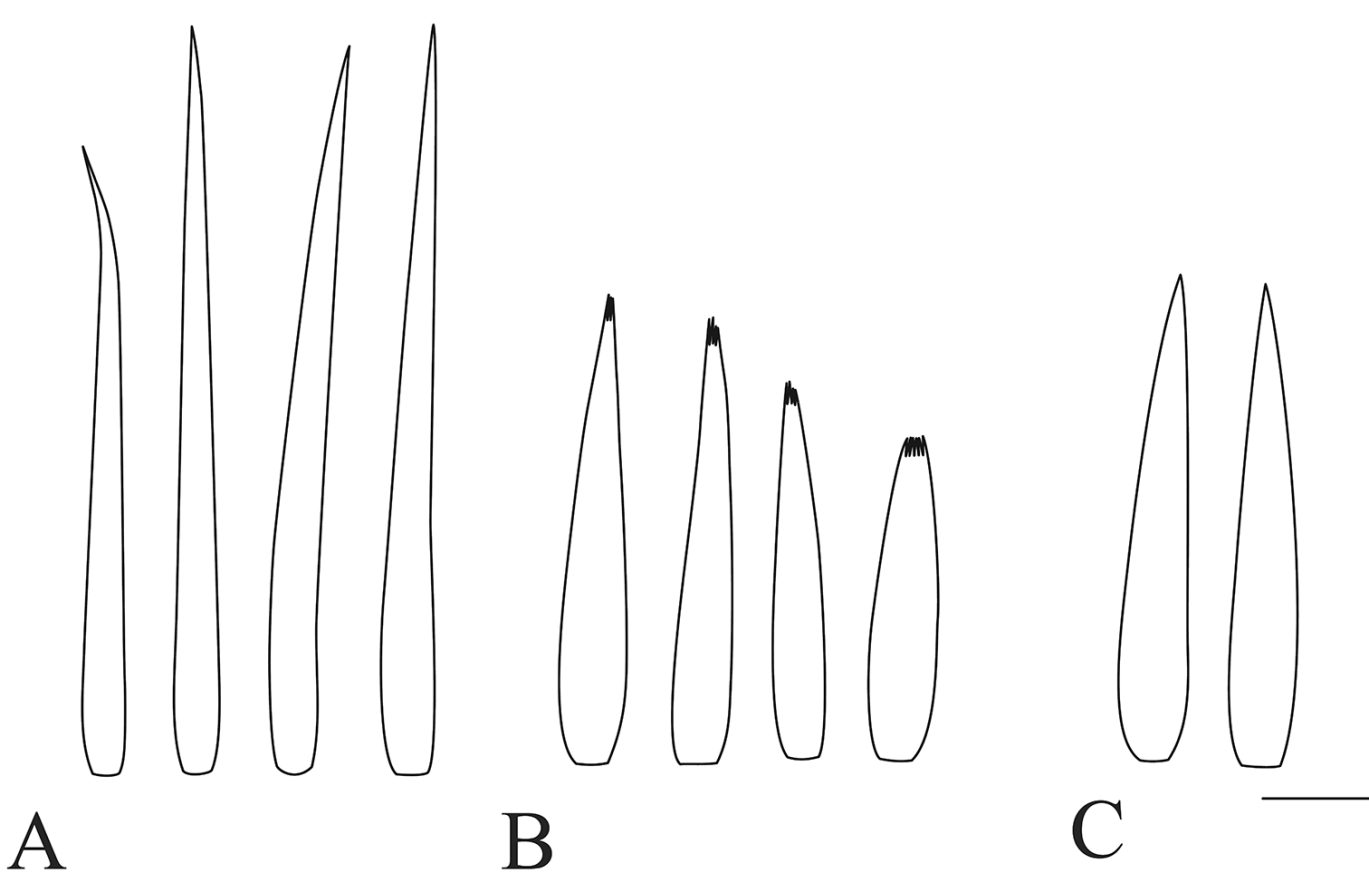
Forefemur transverse row of setae. **A***Dudgeodes bauernfeindi* sp. nov. **B***D. pangantihoni* sp. nov. **C***D. tabang* sp. nov. Scale bar: 20 μm.

The transverse row of setae on the fore femur is generally useful. The shape can be wider, similar to *Dudgeodes pescadori* (Sartori et al. 2008: fig. 72), or narrower as in *D. bauernfeindi* sp. nov. (Fig. 14A). The apex of which can be combed (Fig. 14B), or bluntly pointed (Fig. 14C) as in *D. bauernfeindi* sp. nov. and *D. tabang* sp. nov. However, this transverse row is usually covered with debris and the examination of such characters may be challenging sometimes.

Second, dorsal surface, outer margin and inner margin of femora are mostly sparsely covered with short, thick, apically combed setae (Martynov et al. 2016: fig. 19) but may be missing as in *Dudgeodes bauernfeindi* sp. nov. A bunch of thin setae may also be present on the outer margin as in *D. pangantihoni* sp. nov. and *D. freitagi* sp. nov.

The tarsal claws offer interesting differences. The number of blunt teeth medially varies from three to five within conspecific samples; within the same individual, the hind tarsal claw may have +1 medial tooth. However, the number of subapical teeth seems to be a more stable character. They are absent in *Dudgeodes freitagi* sp. nov., *D. luntian* sp. nov., *D. pangantihoni* sp. nov., and *D. pescadori*, a single tooth is present in *D. tabang* sp. nov., or two are present in *D. bauernfeindi* sp. nov. and *D. vonrinteleni* sp. nov. The fore tarsal claw is more prone to wear and tear; the number of subapical teeth of the fore tarsal claw sometimes varies in the same specimen and among conspecific samples in contrast to the middle and hind tarsal claw of the same individual with usually stable number of teeth. Hence it is recommended to use these when the fore tarsal claw offers a different number. The apex of claws has 3–5 thin setae laterally, but this character is not diagnostic.

Other ornamentations in the legs are generally similar and not diagnostic. The forefemur outer margin is covered by thick and long setae meeting the transverse row. The inner margin is covered with a short row of long and thin setae proximally reaching distally to the transverse row. Middle and hind femora are similar and more slender than the forefemur. Both middle and hind femur outer and inner margins are covered with row of long pointed thick setae, the inner margin begins at the dorsal surface. The foretibia outer margin has medium-sized thin setae, the inner margin with few apically pointed short thick setae, and the dorsal surface has regular row of long thick setae. Middle and hind tibiae outer margin and dorsal surface with regular row of long thick setae; inner margin with few short apically pointed thick setae.

Abdomen

The posterolateral projections are generally useful in differentiating species and are best examined from the ventral side. Careful consideration for ranking intact female specimens (i.e., not mounted on slides) should be made as egg-filled abdomen may give an illusion of the presence of posterolateral projection. The rows of long thick setae on the lateral margin of tergites IV–X may also give such illusion. Here, we consider the projection well developed when the length of the projection is equal to the corresponding segment length, moderately developed when it is half the length of the segment, and slightly marked when it is less than half length but still present.

Median tubercles are also common. All tergites have median tubercles. We consider them well developed when the length of the tubercles is half of the corresponding tergite length, moderately developed when they are 1/3 of the tergite length, and slightly developed when they are smaller than the latter but still present. All median tubercles are densely covered with apically rounded thick setae with oval margins (sensu Martynov et al. 2016: fig. 14).

Ornamentations on the tergites I–III are generally the same as mentioned in Martynov et al. (2016), and likely similar to other *Dudgeodes*. The surface and posterior margin of tergites I–III bear few long thin setae, more abundant in the submedian area. These are sometimes absent on conspecific samples probably due to slide preparation procedure. Tergites IV–VI ornamentations of most species are comparable to figure 15 in Martynov et al. (2016) but are different in *D. pescadori*, *D. tabang* sp. nov., and *D. vonrinteleni* sp. nov. as they lack elongated forked setae on the posterior margin. Within these three tergites, ornamentations are more abundant in tergite VI. Ornamentations on tergites VII–X of most species are as depicted in Martynov et al. (2016: fig. 16) but are different in *D. bauernfeindi* sp. nov. In contrast to *D. romani*, tergite X still has posterior margin ornamentations like in tergite IX, but less abundant and often absent in conspecific samples probably due to slide preparation procedure.

**Figure 15.**
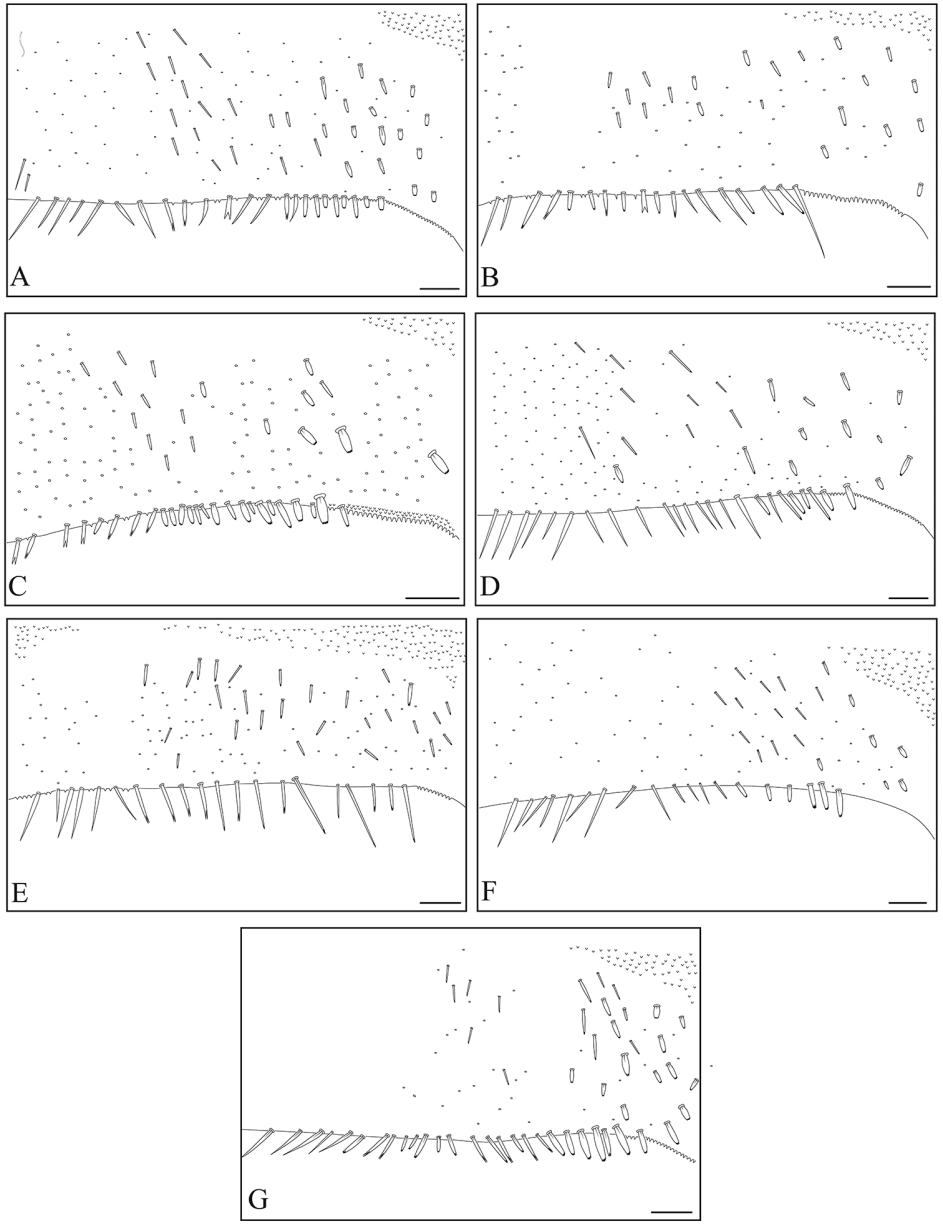
Tergite VI ornamentations of Philippine *Dudgeodes* species. **A***D. pangantihoni* sp. nov. **B***D. luntian* sp. nov. **C***D. vonrinteleni* sp. nov. **D***D. pescadori***E***D. bauernfeindi* sp. nov. **F***D. tabang* sp. nov. **G***D. freitagi* sp. nov. Scale bar: 50 μm.

**Figure 16.**
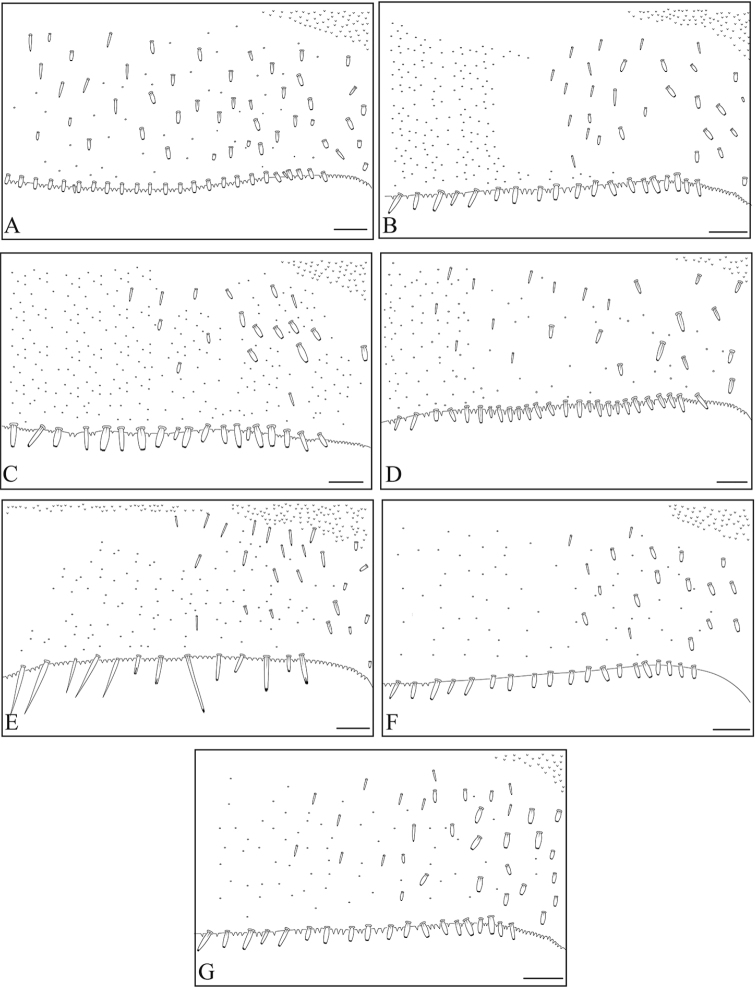
Tergite VII ornamentations of Philippine *Dudgeodes* species. **A***D. pangantihoni* sp. nov. **B***D. luntian* sp. nov. **C***D. vonrinteleni* sp. nov. **D***D. pescadori***E***D. bauernfeindi* sp. nov. **F***D. tabang* sp. nov. **G***D. freitagi* sp. nov. Scale bar: 50 μm.

Aside from the ornamentation with setae, narrow slender pointed teeth are also present on the posterior tergite margin and may help in differentiating the Philippine representatives. These may be regularly present across the entire posterior margin (Fig. 16A–E, G) or concentrated only on the certain portions (i.e., median area near the tubercle, lateral area near the posterolateral projection) (Figs 15A–G, 16F). We do recommend examining the entire length of the margin in contrast to the submedian area as in Martynov et al. (2016: figs 15, 16).

Cerci exhibit valuable characters. A brownish band (sensu Anbalagan et al. 2015, partly seen on fig. 1b) may be present as in *Dudgeodes bauernfeindi* sp. nov., *D. freitagi* sp. nov., *D. luntian* sp. nov., *D. pescadori* and *D. tabang* sp. nov., or absent as in *D. pangantihoni* sp. nov. and *D. vonrinteleni* sp. nov. This brownish band appears to be not affected by preservation condition as observed in *D. pescadori* paratypes collected way back in 1977 (Fig. 8). Every segment has setae. However, the length of the longest setae varies and is significant in differentiating species. The longest setae on the basal segments may vary from the distal ones. The species treated here may have setae with length less than half, half, subequal or greater than segment length. The paracercus is always absent in *Dudgeodes* (Figs 2A, 3A, 4A, 5A, 6A, 7A).

The gills are genus-typical for *Dudgeodes* and are generally less informative in differentiating Philippine species. Gills are present on segments II–V. Gill II with operculate dorsal lamella which is oval and has entire margin, i.e., not incised medially; gills III–V with ventral lamella incised medially; gill V lacks lobes on the ventral lamella .

### Descriptions of new species

#### Dudgeodes bauernfeindi

Taxon classificationAnimaliaEphemeropteraTeloganodidae

Garces & Sartori sp. nov.

EB2674C8-A442-5D58-A6AC-189CD97ED1C3

http://zoobank.org/186ED910-579F-43A6-A835-8B55EA36743C

Figures 2A–C , 12D , 14A , 15E , 16E

##### Material.

***Holotype***: Philippines • 1 male nymph; Negros Oriental, Valencia, Casaroro River downstream; 09°18'N, 123°14'E; c. 150 m a.s.l.; 1 Sep. 2019; leg. Garces; GenBank: MN853779; PNM: EPH 356/PNM 13683 in ethanol. ***Paratypes***: Philippines • 2 nymphs; same collection data as holotype; GenBank: MN853780, MN853785; AdMU: EPH 155 in ethanol, EPH 357 on slide • 3 nymphs; Negros Oriental, Valencia, Apolong, Casaroro River upstream; 09°17'N, 123°13'E; 470 m a.s.l.; 1 Sep. 2019; leg. Garces; GenBank: MN853783, MN853782, MN853781; AdMU: EPH 160 in ethanol, ZSM: EPH 158 in ethanol, EPH 157 on slide • 1 nymph; Negros Oriental, Valencia, Malaunay; 09°18'22"N, 123°10'04"E; c. 470 m a.s.l.; 1 Sep. 2019; GenBank: MN853779; AdMU: EPH 150 on slide.

##### Diagnosis.

*Dudgeodes bauernfeindi* sp. nov. can be distinguished from other Oriental *Dudgeodes* by the combination of the following characters: (1) antenna length subequal to head width, (2) dorsal part of male eyes brown, (3) two prothorax tubercles, (4) simple mesothorax marginal setae, (5) two tarsal claw subapical teeth, (6) narrow teeth on median and lateral area of posterior margin tergite VI, (7) tergite VII posterior margin teeth present on the entire length, (8) longest setae on cerci basal segments more than half of the corresponding segment (9) longest setae on cerci distal segments half to the corresponding segments, and (10) presence of brownish band every four segments of the cerci. *Dudgeodes bauernfeindi* sp. nov. can be differentiated from *D. luntian* sp. nov. found also in Negros island by the length of antenna relative to head width, color of dorsal part of male eyes, mesothorax marginal setae and tarsal claw subapical teeth.

##### Description.

Mature nymph. Body length ♂ 5.0–5.2 mm; cerci 0.9–1.0 times body length.

***Head***: Antennae 0.9–1.0 times head width, flagellum with 16–18 segments. Dorsal part of male eyes brown (Fig. 2A). Labrum wide, ca. 2.3–2.5 times wider than long, concave in central part of anterior margin. Labial palp segment III elongated, 2.8–3.0 times as long as wide at base.

***Thorax***: Pronotum (Fig. 2B) with four flat tubercles. Mesonotum (Fig. 2B) without tubercle; outer margin with regular row of simple setae.

***Legs***: Forefemur dilated, ca. 1.5–1.6 times longer than wide; transverse row of long and bluntly pointed setae (Fig. 14A) across dorsal face; dorsal surface sparsely covered with long thin simple setae. Fore tarsal claw hooked, bearing four blunt teeth medially and two teeth subapically. Middle and hind femora ca. 2 times longer than wide. Middle and hind tarsal claw hooked, bearing four to five blunt teeth medially and two teeth subapically.

***Abdomen***: Tergite with median tubercles (Fig. 2B) slightly developed on segments I and II, moderately developed on segment III and IX–X, well developed on IV–IX. Posterolateral projections (Fig. 2C) slightly developed on segments II–IV, moderately developed on V–IX.

Tergites IV–X (Figs 15E, 16E) surface covered with short thin setae, thick setae with feathered apex and apically rounded feathered thick setae; posterior margin with long pointed thick setae, elongated forked thick setae and long setae with feathered apex. Narrow slender teeth present on posterior margin median area of tergites I–V, posterior margin median and lateral areas of tergite VI (Fig. 15E), and across entire posterior margin of tergites VII–X (Fig. 16E).

Cerci with apically blunt thick setae every segment; longest setae on basal segments more than half of corresponding segment; longest setae on distal segments half of corresponding segments. Brownish band present every four segments (Fig. 2A).

##### Etymology.

The species is named after Dr. Ernst Bauernfeind (Vienna), one of the mentors of the first author, for his kindness and outstanding contribution to mayfly taxonomy.

##### Distribution and biology.

*Dudgeodes bauernfeindi* sp. nov. is so far only known from Negros island, Philippines. All material was collected at altitudes of 150–500 m a.s.l. mostly surrounded by secondary vegetation, rarely secondary forest, with few houses in some distance from the river bed.

#### Dudgeodes freitagi

Taxon classificationAnimaliaEphemeropteraTeloganodidae

Garces & Sartori sp. nov.

77CA95EA-491C-5346-990C-E6E1697A6DAE

http://zoobank.org/78B31301-3181-4FDD-BECE-D4ABAD6CEFA2

Figures 3A–C , 12F , 15G , 16G

##### Material.

***Holotype***: Philippines • 1 female nymph; Luzon, Laguna, Samil River; 14°08'N, 121°31'E; 370 m a.s.l.; 27 June 2018; BIO-PHIL exped.; GenBank: MN853790; PNM: EPH 191/PNM 13685 in ethanol. ***Paratypes***: Philippines • 1 nymph; same collection data as holotype; GenBank: MN853789; ZSM: EPH 184 on slide • 3 nymphs; Luzon, Batangas, Lobo, Lobo River; c. 13°40'43"N, 121°15'04"E; c. 38 m a.s.l.; 10 Nov. 1996; leg. Mendoza; GenBank: MN853788, MN853786, MN853787; AdMU: EPH 148 on slide, EPH 146 and EPH 147 in ethanol • 1 nymph; Luzon, Bulacan, 14km E San Miguel, Biak na Bato NP, river 300m upstream B. Panici Cave; 15°06'N, 121°06'E; c. 100 m a.s.l.; 4 Oct. 1995; GenBank: MN853791; AdMU: EPH 238 on slide • 2 nymphs; Luzon, Subic BMA area, Jadjad River; 14°48'59"N, 120°20'31"E; 80 m a.s.l.; 1 July 2017; leg. Freitag; GenBank: MN853792, MN853793; AdMU: EPH 245 and EPH 246 on slides.

##### Diagnosis.

*Dudgeodes freitagi* sp. nov. can be distinguished from other Oriental *Dudgeodes* by the combination of the following characters: (1) antenna length longer than head width, (2) dorsal part of male eyes blackish, (3) six prothorax tubercles, (4) two mesothorax tubercles, (5) no tarsal claw subapical teeth, (6) narrow teeth on median area of posterior margin tergite VI, (7) narrow teeth on entire area of posterior margin tergite VII, (8) longest setae on cerci basal segments half of the corresponding segment (9) longest setae on cerci distal segments greater than the corresponding segments, and (10) presence of brownish band every four segments of the cerci. Among the species found on Luzon island, *Dudgeodes freitagi* sp. nov. closely resembles *D. pescadori* and *D. luntian* sp. nov. but can be separated by the combination of the following characters: (1) antenna length longer than head width, (2) six prothorax tubercles, (3) two mesothorax tubercles, (4) five to six tarsal medial teeth, (5) narrow teeth on median area of posterior margin of tergite VI, (6) longest setae on cerci basal segments half of the corresponding segment, (7) longest setae on cerci distal segments greater than the corresponding segments, (8) median tubercles moderately developed on segments I and II and VIII–X, well developed on segments III–VII, and (9) posterolateral projection slightly developed on V–VIII.

##### Description.

Mature nymph. Body length ♂ 4.8–5.2 mm, ♀ 5.7–5.9 mm; cerci 0.8–0.9 times body length.

***Head***: Antennae 1.1–1.2 times head width, flagellum with 16 or 17 segments. Dorsal part of male eyes blackish (Fig. 3A). Labrum wide, ca. 2.2–2.3 times wider than long, slightly concave in central part of anterior margin. Labial segment III elongated, 2.7–3.3 times as long as wide at base.

***Thorax***: Pronotum (Fig. 3B) with six small and round tubercles. Mesonotum (Fig. 3B) with two small and round tubercles; outer margin with regular row of forked setae.

***Legs***: Forefemur dilated, ca. 1.5–1.7 times longer than wide; transverse row of long and apically combed setae across dorsal face; dorsal surface, outer and inner margin sparsely with short thick setae and thin setae in solitary and in bunches. Fore tarsal claw hooked, bearing five blunt teeth medially and no tooth subapically. Middle and hind femora ca. 2 times longer than wide. Middle and hind tarsal claw hooked, bearing four to five blunt teeth medially and no tooth subapically.

***Abdomen***: Tergite (Fig. 3B) with median tubercles moderately developed on segments I–II and VIII–X, well developed on segments III–VII. Posterolateral projections (Fig. 3C) absent on segments II–III, slightly developed on IV–IX.

Tergites IV–VI (Fig. 15G) surface covered with short thin setae, thick setae with feathered apex and apically rounded feathered thick setae; posterior margin with long pointed thick setae, forked thick setae and thick setae with feathered apex and apically rounded feathered thick setae. Tergites VII–X (Fig. 16G) surface and posterior margin with short thin setae, thick setae with feathered apex and apically rounded feathered thick setae. Narrow slender teeth present on posterior margin median area of tergites I–VI (Fig. 15G) and across entire posterior margin of tergites VII–X (Fig. 16G).

Cerci with apically blunt thick setae every segment; longest setae on basal segments half of corresponding segment; longest setae on distal segments longer than corresponding segment length. Brownish band present every four segments (Fig. 3A).

##### Etymology.

The species is named after Professor Hendrik Freitag (Philippines/Germany), the collector of some material, for being a great mentor to freshwater entomology students in the Philippines. His experience and passion motivated the first author to continue pursuing research on aquatic insects.

##### Distribution and biology.

**Figure 17.**
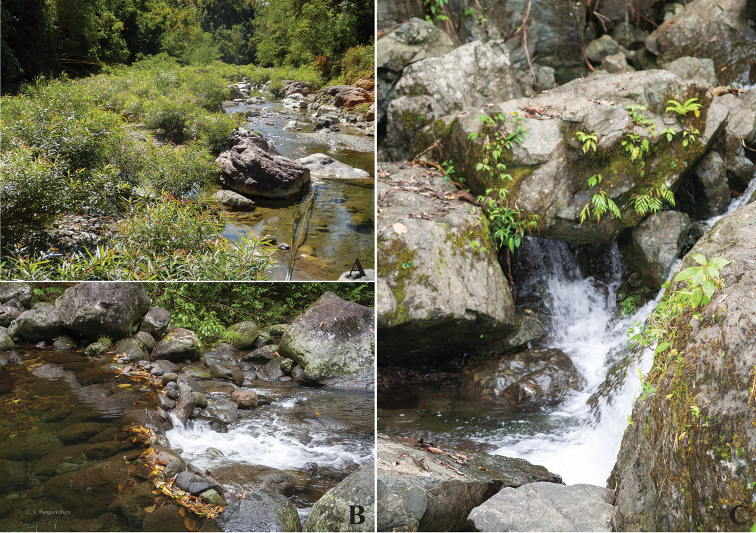
Collection sites of Philippine *Dudgeodes* species. **A** Jadjad River, Luzon (*D. freitagi* sp. nov.) **B** Mimpis River, Luzon (*D. vonrinteleni* sp. nov.) **C** Payas River, Luzon (*D. pangantihoni* sp. nov.).

*Dudgeodes freitagi* sp. nov. is so far only known from the Luzon island. All material was collected at altitudes of 10–400 m a.s.l. surrounded by secondary vegetation or farmland, with few houses in some distance from the river bed (Fig. 17A).

#### Dudgeodes luntian

Taxon classificationAnimaliaEphemeropteraTeloganodidae

Garces & Sartori sp. nov.

5E2438C5-BC07-51F7-8AE5-14893917B3E8

http://zoobank.org/D13F5B81-5682-491A-AF2A-9D25D99552C3

Figures 4A–C , 9A–H , 12B , 15B , 16B

##### Material.

***Holotype***: Philippines • 1 mature male nymph; Luzon, Laguna, Pangil, Brgy. Balian, Pangil River; 14°24'55"N, 121°29'36"E; c. 200 m a.s.l.; 18 Aug. 2018; leg. Amarga; GenBank: MN853796; PNM: EPH 137/PNM 13681 in ethanol. ***Paratypes***: Philippines • 1 nymph; same collection data as holotype; GenBank: MN853795; AdMU: EPH 136 on slide • 1 nymph; Oriental Mindoro, Roxas, Brgy. San Vicente, lower reach of Taugad River; 12°37.30'N, 121°22.97'E; 140 m a.s.l.; 8 Dec. 2018; leg. Garces; GenBank: MN853794; AdMU: EPH 127 on slide • 2 nymphs; Luzon, Laguna, Samil River; 14°08'N, 121°31'E; 370 m a.s.l.; 27 June 2018; BIO-PHIL exped.; GenBank: MN853805, MN853806; ZSM: EPH 185 in ethanol, EPH 186 on slide • 1 nymph; Camarines Sur, Lupi, Brgy. Sooc, Sooc River, Bicol National Park; c. 13°52'28"N, 122°56'38"E; 90 m a.s.l.; 20 Aug. 1996; leg. Mendoza; GenBank: MN853804; AdMU: EPH 173 in ethanol • 1 nymph; Luzon, Subic BMA, Triboa River, downstream dam; 14°46'22"N, 120°17'50"E; 60 m a.s.l.; 14 Apr. 2018; leg. Freitag; GenBank: MN853808; AdMU: EPH 247 on slide • 4 nymphs; Negros Oriental, Valencia, Casaroro River downstream; 09°18'N, 123°14'E; c. 150 m a.s.l.; 2019; leg. Garces; GenBank: MN853802, MN853803, MN853800, MN853801; AdMU: EPH 156 on slide, EPH 159 in ethanol, ZSM: EPH 153 on slide, EPH 154 in ethanol • 1 nymph; Negros Oriental, Valencia, Malaunay, small tributary; 09°18'17"N, 123°10'07"E; c. 480 m a.s.l.; 2019; leg. Garces; GenBank: MN85379; AdMU: EPH 149 on slide • 3 nymphs; Negros Oriental, Valencia, Malaunay, small river; 09°18'17"N, 123°10'07"E; c. 470 m a.s.l.; 2019; leg. Garces; GenBank: MN853799, MN853807, MN853798; AdMU: EPH 152 and EPH 239 in ethanol, ZSM: EPH 151 on slide.

##### Diagnosis.

*Dudgeodes luntian* sp. nov. can be distinguished from other Oriental *Dudgeodes* by the combination of the following characters: (1) antenna length shorter than head width, (2) dorsal part of male eyes blackish, (3) four prothorax tubercles, (4) two mesothorax tubercles, (5) no tarsal claw subapical teeth, (6) narrow teeth on median and lateral area of posterior margin of tergite VI, (7) tergite VII posterior margin teeth present on the entire length, (8) longest setae on cerci basal segments more than half of the corresponding segment (9) longest setae on cerci distal segments subequal to the corresponding segments, and (10) presence of brownish band every four segments of the cerci. Among the other *Dudgeodes* species found on Luzon and Negros islands, *D. luntian* sp. nov. closely resembles *D. pescadori* and *D. freitagi* sp. nov. but can be separated by the combination of the following characters: (1) antenna length shorter than head width, (2) four prothorax tubercles, (3) two mesothorax tubercles, (4) five to six tarsal medial teeth, (5) narrow teeth on median and lateral area of posterior margin tergite VI, (6) longest setae on cerci basal segments more than half of the corresponding segment (7) longest setae on cerci distal segments subequal to the corresponding segments, (8) median tubercles slightly developed on tergite segments I, II, and X, moderately developed on segments III and IX, and well developed on segment VIII, and (9) posterolateral projection on segments V–VIII slightly developed and segment IX moderately developed.

##### Description.

Mature nymph. Body length ♂ 5.6–5.8 mm, ♀ 6.3 mm; cerci 0.9–1.0 times body length.

***Head***: Antennae 0.7–0.8 times head width, flagellum with 15–17 segments. Dorsal part of male eyes blackish (Fig. 9A–H). Labrum wide, ca. 2.3–2.4 times wider than long, slightly concave in central part of anterior margin. Labial palp segment III elongated, 2.8–3.2 times as long as wide at base.

***Thorax***: Pronotum (Fig. 4B) with four small and round tubercles. Mesonotum (Fig. 4B) with two round tubercles; outer margin with regular row of forked thick setae.

***Legs***: Forefemur dilated, ca. 1.5–1.7 times longer than wide; transverse row of long and apically combed setae across dorsal face; dorsal surface, outer and inner margin sparsely with short thick setae. Fore tarsal claw hooked, bearing five blunt teeth medially and no teeth subapically. Middle and hind femora ca. 2.1–2.2 times longer than wide. Middle and hind tarsal claw hooked, bearing six blunt teeth medially and no tooth subapically.

***Abdomen***: Tergite with median tubercles (Fig. 4B) slightly developed on segments I, II, and X, moderately developed on segments III and IX, and well developed on segments IV–VIII. No posterolateral projections (Fig. 4C) on segments II and III, slightly developed on segments IV–VIII, and moderately developed on segment IX.

Tergites IV–VI (Fig. 15B) surface covered with short thin setae, thick setae with feathered apex and apically rounded feathered thick setae; posterior margin with long pointed thick setae, forked robust thick setae and thick setae with feathered apex and apically rounded feathered thick setae. Tergites VII–X (Fig. 16B) surface and posterior margin with short thin setae, thick setae with feathered apex and apically rounded feathered thick setae. Narrow slender teeth present on posterior margin median area of tergites I–V, posterior margin median and lateral area of tergite VI (Fig. 15B), and across entire posterior margin of tergites VII–X (Fig. 16B).

Cerci with apically blunt thick setae every segment; longest setae on basal segments more than half of corresponding segment; longest setae on distal segments subequal to corresponding segments. Brownish band present every four segments (Fig. 4A).

##### Etymology.

The species is named after the Filipino word ‘luntian’ meaning green, which perfectly describes the localities where this species is found.

##### Distribution and biology.

*Dudgeodes luntian* sp. nov. is so far only known from Luzon, Mindoro and Negros islands, Philippines. All material was collected at altitudes of 20–500 m a.s.l. mostly surrounded by secondary vegetation, rarely secondary forest, with few houses and farmland in some distance from the river bed. Detailed ecological information of the Mindoro locality is described in Garces et al. (2018).

#### Dudgeodes pangantihoni

Taxon classificationAnimaliaEphemeropteraTeloganodidae

Garces & Sartori sp. nov.

4FC84F1B-5348-5B11-8881-E4A2855F028E

http://zoobank.org/95FC37E7-EF2D-4337-9D86-426E74259446

Figures 5A–C , 10A–H , 11A–E , 12A , 13A–D , 14B , 15A , 16A

##### Material.

***Holotype***: Philippines • 1 mature male nymph; Camiguin, Sagay, Bonbon, lower Binangawan River; 09°06'39"N, 124°43'45"E; 26 m a.s.l.; 9 Dec. 2018; leg. Freitag; GenBank: MN853821; PNM: EPH 220/PNM 13680 in ethanol. ***Paratypes***: Philippines • 4 nymphs; same collection data as holotype; GenBank: MN853809, MN853811, MN853822, MN853812; AdMU: EPH 163, EPH 166, EPH 221 on slides, ZSM: EPH 168 on slide • 2 nymphs; Mindanao, Agusan del Sur, San Francisco, Bayogan, Tagkunayan Creek; 8°28'N, 125°59'E; 120 m a.s.l.; 5 Feb. 1998; leg. Mendoza; GenBank: MN853813, MN853810; ZSM: EPH 169 in ethanol, EPH 165 on slide • 2 nymphs; Mindanao, Bukidnon, Cagayan River, Tignapoloan Falls; 8°15'10"N, 124°34'51"E; c.270 m a.s.l.; 19 June 1997; leg. Mendoza; GenBank: MN853815, MN853814; AdMU: EPH 208 in ethanol, ZSM: EPH 207 on slide • 1 nymph; Mindanao, Bukidnon, Cagayan River; 8°15'07"N, 124°34'56"E; c.250 m a.s.l.; 19 June 1997; leg. Mendoza; GenBank: MN853820; AdMU: EPH 213 on slide • 3 nymphs; Mindanao, Agusan N, Cabadbaran, Del Pilar, Payas River; 9°11'34"N, 125°36'34"E; c.660 m a.s.l.; 23 June 2018; leg. Pangantihon; GenBank: MN853816, MN853817, MN853818; AdMU: EPH 209 in ethanol, AdMU EPH 210 on slide, ZSM: EPH 211 in ethanol • 1 nymph; Mindanao, Bukidnon, CEDAR; 8°15'10"N, 125°02'07"E; 760 m a.s.l.; 18 Nov. 1997; leg. Mendoza; GenBank: MN853819; AdMU: EPH 212 on slide.

##### Diagnosis.

*Dudgeodes pangantihoni* sp. nov. can be distinguished from other Oriental *Dudgeodes* by the combination of the following characters: (1) antenna length subequal to head width, (2) dorsal part of male eyes brown, (3) six prothorax tubercles, (4) two mesothorax tubercles, (5) no tarsal claw subapical teeth, (6) tergite VI posterior margin teeth diminishing distally, (7) tergite VII posterior margin teeth present on the entire length, (8) longest setae on cerci basal segments more than half of the corresponding segment, (9) longest setae on cerci distal segments subequal to the corresponding segments, and (10) absence of brownish band on the cerci. *Dudgeodes pangantihoni* sp. nov. is most similar to *D. celebensis* from which it can be separated by the combination of the following characters: (1) dorsal part of male eyes brown, (2) tergite VII posterior margin teeth present on the entire length, (3) moderately developed median tubercles of tergites III–IX, (4) no posterolateral projections on segments III and IV, (5) moderately developed posterolateral projections on segments II and segments VII–IX, and (6) slightly marked posterolateral projections on segments V and VI.

##### Description.

Mature nymph. Body length ♂ 4.7–5.2, ♀5.7 mm; cerci 0.8–0.9 times body length.

***Head***: Antennae 0.9–1.0 times head width, flagellum with 16 or 17 segments. Dorsal part of male eyes brown (Fig. 10A–H). Labrum wide, ca. 2.0–2.2 times wider than long, slightly concave in central part of anterior margin. Labial palp segment III elongated, 3.0–3.3 times as long as wide at base.

***Thorax***: Pronotum (Fig. 13A–D) with six small and round tubercles. Mesonotum (Fig. 13A–D) with two small and round tubercles; outer margin with regular row of forked thick setae.

***Legs***: Forefemur dilated, ca. 1.5–1.7 times longer than wide; transverse row of long and apically combed setae (Fig. 14B) across dorsal face; dorsal surface, outer and inner margin sparsely covered with short thick apically combed setae and thin setae solitary and in bunches. Fore tarsal claw hooked, bearing four blunt teeth medially and no tooth subapically. Middle and hind femora ca. 2 times longer than wide. Middle and hind tarsal claw hooked, bearing four blunt teeth medially and no tooth subapically.

***Abdomen***: Tergite with median tubercles (Fig. 5B) moderately developed on segments I–IX, slightly developed on segment X. No posterolateral projections (Fig. 5C) on segments III and IV; moderately developed on segment II and segments VII–IX; slightly marked on segment V and VI.

Tergites IV–VI (Fig. 15A) surface covered with short thin setae, thick setae with feathered apex and apically rounded feathered thick setae; posterior margin with long pointed thick setae, forked robust thick setae and thick setae with feathered apex and apically rounded feathered thick setae. Tergites VII–X (Fig. 16A) surface and posterior margin with short thin setae, thick setae with feathered apex and apically rounded feathered thick setae. Narrow slender teeth present on posterior margin of tergites I–VI diminishing laterally (Fig. 15A) and across entire posterior margin of tergites VII–X (Fig. 16A).

Cerci with apically blunt thick setae every segment; longest setae on basal segments more than half of corresponding segment; longest setae on distal segments subequal to corresponding segments. Brownish band absent (Fig. 5A).

##### Etymology.

The species is named after Clister V. Pangantihon, the collector of some material and project assistant of the Biodiversity Laboratory, AdMU.

##### Distribution and biology.

*Dudgeodes pangantihoni* sp. nov. is so far only known from Northern Mindanao (Fig. 17C) and Camiguin island, Philippines. All material was collected at altitudes of 20–600 m a.s.l. surrounded by secondary vegetation, rarely secondary forest. Nymphs from Camiguin island were collected in small lowland river surrounded by secondary vegetation, coconut farmland.

#### Dudgeodes tabang

Taxon classificationAnimaliaEphemeropteraTeloganodidae

Garces & Sartori sp. nov.

9A6807E8-9377-5911-80F1-E38D41E48959

http://zoobank.org/3C50C564-4055-4309-B89F-30A1DAB5F6B6

Figures 6A–C , 12E , 14C , 15F , 16F

##### Material.

***Holotype***: Philippines • 1 mature female nymph; Leyte, Ormoc, Dolores, Lake Danao outflow creek; 11°04'37"N, 124°41'46"E; 650 m a.s.l.; 26 Sep. 2019; leg. Garces; GenBank: MN853829; PNM: EPH 194/PNM 13684 in ethanol. ***Paratypes***: Philippines • 1 nymph; same collection data as holotype; GenBank: MN853828; AdMU: EPH 193 on slide • 5 nymphs; Leyte, Ormoc, Dolores, Lake Danao tributary; 11°03'55"N, 124°42'17"E; 650m asl.; 26 Sep. 2019; leg. Garces; GenBank: MN853830, MN853832, MN853834, MN853831, MN853833; AdMU: EPH 331, EPH 333, EPH 335 on slides, EPH 332 in ethanol, ZSM: EPH 334 in ethanol.

##### Diagnosis.

*Dudgeodes tabang* sp. nov. can be distinguished from other Oriental *Dudgeodes* by the combination of the following characters: (1) antenna length longer than head width, (2) dorsal part of male eyes brown, (3) four prothorax tubercles, (4) pointed forefemur transverse setae apex, (5) one tarsal claw subapical teeth, (6) no teeth on tergite VI posterior margin, (7) narrow teeth on lateral area of posterior margin tergite VII, (8) longest setae on cerci basal segments half of the corresponding segment (9) longest setae on cerci distal segments greater than the corresponding segments, and (10) presence of brownish band every four segments of the cerci. *Dudgeodes tabang* sp. nov. resembles *D. hutanis* based on size, antenna length and thorax tubercles but can be separated by color of dorsal part of male eyes, number of subapical tarsal claw teeth and presence of brownish band on cerci.

##### Description.

Mature nymph. Body length ♂ 4.6–5.0 mm; cerci 0.8–0.9 times body length.

***Head***: Antennae 1.0–1.2 times head width, flagellum with 16–18 segments. Dorsal part of male eyes brown (Fig. 6A). Labrum wide, ca. 2.4–2.6 times wider than long, slightly concave in central part of anterior margin. Labial palp segment III elongated, 3.2–3.4 times as long as wide at base.

***Thorax***: Pronotum (Fig. 6B) with four round tubercles. Mesonotum (Fig. 6B) without tubercle; outer margin with regular row of forked thick setae.

***Legs***: Forefemur dilated, ca. 1.4–1.6 times longer than wide; transverse row of pointed thick setae (Fig. 14C) across dorsal face; dorsal surface, outer and inner margin sparsely with short thick setae. Fore tarsal claw hooked, bearing four blunt teeth medially and one tooth subapically. Middle and hind femora ca. 2 times longer than wide. Middle and hind tarsal claw hooked, bearing four blunt teeth medially and one tooth subapically.

***Abdomen***: Tergite with median tubercles (Fig. 6B) moderately developed on segments I and II, well developed on segments III to X. No posterolateral projections (Fig. 6C) on segments II and III, slightly developed on segments IV and V, moderately developed on segments VI–IX.

Tergites IV–VI (Fig. 15F) surface covered with short thin setae, thick setae with feathered apex and apically rounded feathered thick setae; posterior margin with long pointed thick setae, thick setae with feathered apex and apically rounded feathered thick setae. Tergites VII–X (Fig. 16F) surface and posterior margin with short thin setae, thick setae with feathered apex and apically rounded feathered thick setae. Narrow slender teeth absent on posterior margin of tergites I–VI (Fig. 15F), present in posterior margin lateral area of tergite VII (Fig. 16F), and present across entire posterior margin of tergites VIII–X.

Cerci with apically blunt thick setae every segment; longest setae on basal segments half of corresponding segment; longest setae on distal segments more than half of corresponding segments. Brownish band present every four segments (Fig. 6A).

##### Etymology.

The species is named after the words *tab-ang* and *tabang* which mean freshwater and help in Cebuano language, respectively. This species alludes to a cry for help from science, private, and government sectors to converge towards a common freshwater conservation goal of enhancing species richness and ecosystem health even, and especially, in human-dominated inland landscapes.

##### Distribution and biology.

*Dudgeodes tabang* sp. nov. is so far only known from Leyte island, Philippines. All material was collected at altitudes of 500–600 m a.s.l. surrounded by secondary vegetation, rarely secondary forest.

#### Dudgeodes vonrinteleni

Taxon classificationAnimaliaEphemeropteraTeloganodidae

Garces & Sartori sp. nov.

C28A0C3E-DB5E-561D-B0DB-BA0D1700B860

http://zoobank.org/EF0B1E10-AB99-4860-94E9-EB63C8561386

Figures 7A–C , 12C , 15C , 16C

##### Material.

***Holotype***: Philippines • 1 mature female nymph; Luzon, Laguna, Majayjay, Bukal, Mimpis River; 14°06'60"N, 121°27'54"E; 425 m a.s.l.; 26 June 2018; BIO-PHIL exped.; GenBank: MN853843; PNM: EPH 345/PNM 13682 in ethanol. ***Paratypes***: Philippines • 6 nymphs; same collection data as holotype; GenBank: MN853835, MN853837, MN853840, MN853841, MN853842, MN853836; AdMU: EPH 180 and EPH 182 on slides, EPH 342, EPH 343, EPH 344 in ethanol, ZSM: EPH 181 on slide • 2 nymphs; Luzon, Laguna, Majayjay, Taytay River; 14°06'42"N, 121°30'19"E; 510 m a.s.l.; 27 June 2018; BIO-PHIL exped.; GenBank: MN853838, MN853839; AdMU: EPH 188 on slide, ZSM: EPH 189 on slide.

##### Diagnosis.

*Dudgeodes vonrinteleni* sp. nov. can be distinguished from other Oriental *Dudgeodes* by the combination of the following characters: (1) antenna length subequal to head width, (2) dorsal part of male eyes blackish, (3) six prothorax tubercles, (4) two mesothorax tubercles, (5) two tarsal claw subapical teeth, (6) narrow teeth on median and lateral area of posterior margin tergite VI, (7) tergite VII posterior margin teeth present on the entire length, (8) longest setae on cerci basal segments more than half of the corresponding segment (9) longest setae on cerci distal segments less than half to the corresponding segments, and (10) absence of brownish band on the cerci. *Dudgeodes vonrinteleni* sp. nov. can be separated from other *Dudgeodes* from Luzon island (*D. pescadori*, *D. luntian* sp. nov., *D. freitagi* sp. nov.) by the number of tarsal claw subapical teeth, longest setae on cerci distal segments less than half to the corresponding segments and absence of brownish band on the cerci. *Dudgeodes vonrinteleni* sp. nov. resembles *D. ulmeri* based on thorax tubercles, tarsal claw and size but can be separated by color of dorsal part of male eyes, apex of forefemur transverse setae, and tergites VI and VII posterior margin teeth presence.

##### Description.

Mature nymph. Body length ♂ 5.1–5.5 mm, ♀ 6.3–6.5 mm; cerci 1.0–1.3 times body length.

***Head***: Antennae 0.9–1.0 times head width, flagellum with 16–18 segments. Dorsal part of male eyes blackish (Fig. 7A). Labrum wide, ca. 2.3–2.5 times wider than long, slightly concave in central part of anterior margin. Labial palp segment III elongated, 3.2–3.6 times as long as wide at base.

***Thorax***: Pronotum (Fig. 7B) with six tubercles, four round and two flat. Mesonotum (Fig. 7B) with two flat tubercles; outer margin with regular row of forked thick setae.

***Legs***: Forefemur dilated, ca. 1.4–1.6 times longer than wide; transverse row of long and apically combed setae across dorsal face; dorsal surface uniformly covered with hair-like setae; dorsal surface, outer and inner margin sparsely with short thick setae. Fore tibia outer margin with middle-sized thin setae; inner margin with few apically pointed thick setae; dorsal surface with regular row of long thick setae. Fore tarsal claw hooked, bearing five blunt teeth medially and two teeth subapically. Middle and hind femora ca. 2 times longer than wide. Middle and hind tarsal claw hooked, bearing four blunt teeth medially and two teeth subapically.

***Abdomen***: Tergite with median tubercles (Fig. 7B) moderately developed on segments I and II, well developed on segments III to X. No posterolateral projections (Fig. 7C) on segments II and III, slightly developed on segments IV and V, moderately developed on segments VI–IX.

Tergites IV–VI (Fig. 15C) surface covered with short thin setae, thick setae with feathered apex and apically rounded feathered thick setae; posterior margin with forked robust thick setae and thick setae with feathered apex and apically rounded feathered thick setae. Tergites VII–X (Fig. 16C) surface and posterior margin with short thin setae, thick setae with feathered apex and apically rounded feathered thick setae. Narrow slender teeth present on posterior margin of tergites I–V diminishing distally, posterior margin median and lateral area of tergite VI (Fig. 15C), and across entire posterior margin of tergites VII–X (Fig. 16C).

Cerci with apically blunt thick setae every segment; longest setae on basal segments more than half of corresponding segment; longest setae on distal segments less than half of corresponding segments. Brownish band absent (Fig. 7A).

##### Etymology.

The species is named after BIO-PHIL project head Dr. Thomas von Rintelen (Berlin) for his continuous support in biodiversity research in Southeast Asia and for the preliminary training of the first author in DNA Taxonomy.

##### Distribution and biology.

*Dudgeodes vonrinteleni* sp. nov. is so far only known from Luzon island. All material was collected at altitudes of 400–500 m a.s.l. surrounded by secondary forest (Fig. 17B).

### Supplementary descriptions of nominal species

#### Dudgeodes celebensis

Taxon classificationAnimaliaEphemeropteraTeloganodidae

Sartori, 2008

4A4B00B8-A888-5E45-9290-DA05BEF55401

##### Material examined.

Indonesia • 7 nymphs, paratype; Sulawesi, vicinity of Manado, Kali Village, site 1 Kali stream, above bridge; 01.41412N, 124.84214E; 8 Dec. 2004; leg. C. Geraci, M. Dien, F. Mirah, D. Lapasi; MZL: 2 on slides GBIFCH00195218 and GBIFCH00195219, 5 in ethanol GBIFCH00195217.

##### Description.

***Thorax***: Mesonotum outer margin with regular row of forked thick setae.

***Abdomen***: Tergites IV–VI surface covered with short thin setae, thick setae with feathered apex and apically rounded feathered thick setae; posterior margin with long pointed thick setae, thick setae with feathered apex and apically rounded feathered thick setae. Tergites VII–X surface and posterior margin with short thin setae, thick setae with feathered apex and apically rounded feathered thick setae. Narrow slender teeth present on posterior margin median area of tergites I–X diminishing distally.

Cerci with apically blunt thick setae on every segment; longest setae on basal segments half of corresponding segment; longest setae on distal segments subequal to corresponding segment. Brownish band absent.

##### Distribution.

Sulawesi.

#### Dudgeodes hutanis

Taxon classificationAnimaliaEphemeropteraTeloganodidae

Sartori, 2008

FB7C1E1C-DA0F-577F-A9A8-E760D0BA497E

##### Material examined.

Indonesia • 1 nymph, paratype; East Kalimantan, Malinau, Seturan (2001-bloc 57), Tamalang, affl. Seturan; 8 Aug. 2000; leg. P. Derleth; MZL: GBIFCH00195312 on slide • 1 nymph, paratype; East Kalimantan, Malinau, Seturan (2000-bloc 43), Temalat (Sungai Guang), affl. Seturan; 16 Aug. 2000; leg. P. Derleth; MZL: GBIFCH00195314 on slide • 8 nymphs, paratypes; East Kalimantan, Malinau Basin, Seturan (2000-petak 43), Temalat (Sungai Guang), trib. Seturan (B0813); 2°59'29"N, 116°33'29"E; 16 March 2001; MZL, in ethanol • 12 nymphs; Langap Sud (1999-petak 24), Rian (B1211); 3°1'40"N, 116°31'5"E; 11 July 2000; MZL, in ethanol.

##### Description.

***Thorax***: Mesonotum outer margin with regular row of forked thick setae.

***Abdomen***: Tergites IV–VI surface covered with short thin setae, thick setae with feathered apex and apically rounded feathered thick setae; posterior margin with long pointed thick setae, thick setae with feathered apex and apically rounded feathered thick setae. Tergites VII–X surface and posterior margin with short thin setae, thick setae with feathered apex and apically rounded feathered thick setae. Narrow slender teeth absent on posterior margin median area of tergites I–VI and present in posterior margin of tergites VII–X diminishing distally.

Cerci with apically blunt thick setae every segment; longest setae on basal segments half of corresponding segment; longest setae on distal segments greater than corresponding segment. Brownish band absent.

##### Distribution.

Borneo.

#### Dudgeodes pescadori

Taxon classificationAnimaliaEphemeropteraTeloganodidae

Sartori, 2008

B5DEEDC8-53EE-5C6F-9771-7ADB67F688F1

Figures 8 , 15D , 16D

##### Material examined.

Philippines • 1 nymph, paratype; Luzon, Laguna Province, Los Baños Province, Mud Springs; 28 Sep. 1969; leg. Reisen; MZL: GBIFCH00195280 on slide • 2 nymphs, paratypes; Luzon, Laguna Province, Los Baños Province, College, Molawin Creek; 28 July. 1977; leg. Realon; MZL: GBIFCH00195281 on slide, AdMU: GBIFCH00195277 on slide • 2 nymphs; same locality as Molawin Creek paratype of *D. pescadori*; GenBank: MN853823, MN853824; AdMU: EPH 130 and EPH 135 on slides • 1 nymph; Luzon, SBMA, small river; 14°50'32"N, 120°21'30"E; 63 m a.s.l.; 01 Feb. 2019; leg. Freitag; GenBank: MN853825; AdMU: EPH 248 on slide.

##### Description.

***Thorax***: Mesonotum outer margin with regular row of forked thick setae.

***Legs***: Forefemur with transverse row of long and pointed thick setae across dorsal face; dorsal surface, outer and inner margin sparsely with short thick setae.

***Abdomen***: Tergite with median tubercles slightly developed on segments I to III, moderately developed on segment IV and IX–X, and well developed on segments V–VIII. No posterolateral projections on segments II and III, slightly developed on segments IV, and moderately developed on segments V–IX. Tergites IV–VI (Fig. 15D) surface covered with short thin setae, thick setae with feathered apex and apically rounded feathered thick setae; posterior margin with long pointed thick setae, thick setae with feathered apex and apically rounded feathered thick setae. Tergites VII–X (Fig. 16D) surface and posterior margin with short thin setae, thick setae with feathered apex and apically rounded feathered thick setae. Narrow slender teeth present on posterior margin median area of tergites I–VI (Fig. 15D) and across entire posterior margin of tergites VII–X (Fig. 16D).

Cerci with apically blunt thick setae every segment; longest setae on basal segments more than half of corresponding segment; longest setae on distal segments half of corresponding segment. Brownish band present every four segments.

##### Distribution.

Luzon Island (Philippines).

#### Dudgeodes stephani

Taxon classificationAnimaliaEphemeropteraTeloganodidae

Sartori, 2008

8FAD81D5-8B59-5FE9-AACE-A3D1B074114C

##### Material examined.

Malaysia • 7 nymphs, paratypes; Sabah, Ranau, Liwagu River at bridge; 335 m a.s.l.; 16 Aug. 1972; leg. W.L. & J.G. Peters; MZL: 2 nymphs on slides GBIFCH00195273 and GBIFCH00195274, 5 in ethanol GBIFCH00195272.

##### Description.

***Thorax***: Mesonotum outer margin with regular row of forked thick setae.

***Abdomen***: Tergites IV–X surface covered with short thin setae, thick setae with feathered apex and apically rounded feathered thick setae; posterior margin with long pointed thick setae, elongated forked thick setae and long setae with feathered apex. Narrow slender teeth present on posterior margin median area of tergites I–X diminishing distally.

Cerci with apically blunt thick setae every segment; longest setae on basal segments half of corresponding segment; longest setae on distal segments subequal to corresponding segment. Brownish band absent.

##### Distribution.

Borneo.

#### Dudgeodes ulmeri

Taxon classificationAnimaliaEphemeropteraTeloganodidae

Sartori, 2008

AAE75A87-CCEB-5677-9645-AAFF41D7F4D3

##### Material examined.

Indonesia • 1 nymph, paratype; Sumatra, stream south of Balige; 5 April 1929; leg. Prof. Feuerborn; MZL: GBIFCH00195208 in ethanol • 1 nymph, paratype; Java, dikes of fishponds at Punten, near Malang; 18 Oct. 1929; leg. Prof. Thienemann; MZL: GBIFCH00195207 in ethanol • 1 nymph; Bali, BLI05, Loc. Baturiti, Desa Antapan; 8°19.344'S, 115°11.606'E; 815 m a.s.l.; 9 October 2009; leg. M Balke & Amran; MZL: GBIFCH00195212 in ethanol • 1 nymph; Sumarat Barat, UN1, Riv. Forest strea, Loc. Universitas Andalas campus; 0°54.666'S, 100°28.379'E; 360 m a.s.l.; 8 November 2011; leg. M. Balke; MZL: GBIFCH00195213 in ethanol.

##### Description.

(see also Sartori 2014).

***Thorax***: Mesonotum outer margin with regular row of forked thick setae.

***Abdomen***: Tergites IV–VI surface covered with short thin setae, thick setae with feathered apex and apically rounded feathered thick setae; posterior margin with long pointed thick setae, elongated forked thick setae and long setae with feathered apex. Tergites VII–X surface and posterior margin with short thin setae, thick setae with feathered apex and apically rounded feathered thick setae. Narrow slender teeth present on posterior margin median area of tergites I–VI, and in posterior margin of tergites VII–X diminishing distally.

Cerci with apically blunt thick setae every segment; longest setae on basal segments more than half of corresponding segment; longest setae on distal segments half of corresponding segment. Brownish band absent.

##### Distribution.

Java, Sumatra.

### Key to known nymphs of Philippine *Dudgeodes*

**Table:** 

1	Tarsal claw without subapical tooth	**2**
–	Tarsal claw with at least 1 subapical tooth	**5**
2	Cerci brownish band absent (Fig. 5A); sterna segment II posterolateral projection present (Fig. 5C); abdominal median tubercle moderately developed on segments V–VII (Fig. 5B); eyes of male nymph brown (Fig. 10A–H)	***D. pangantihoni* sp. nov.**
–	Cerci brownish band present every four segments (Figs 3A, 4A, 8); sterna segment II posterolateral projection absent; abdominal median tubercle well developed on segments V–VII (Figs 3B, 4B); eyes of male nymph blackish (Fig. 9A–H)	**3**
3	Antenna shorter compared to head width; tergite VI lateral posterior margin with narrow teeth (Fig. 15B); abdominal median tubercle on segment X slightly marked (Fig. 4C); longest setae on cerci distal segments subequal to corresponding segment	***D. luntian* sp. nov.**
–	Antenna longer compared to head width; tergite VI lateral posterior margin without narrow teeth (Fig. 15D, G); abdominal median tubercle on segment X moderately developed; longest setae on cerci distal segments half or greater than corresponding segment	**4**
4	Abdominal median tubercle on segment I–II moderately marked, well developed on segments III–IV (Fig. 3B); sterna posterolateral projection slightly marked on segments V–VIII (Fig. 3C)	***D. freitagi* sp. nov.**
–	Abdominal median tubercle on segment I–III slightly marked, moderately developed on segment IV; sterna posterolateral projection moderately developed on segments V–VIII	***D. pescadori***
5	Tarsal claw with 1 developed subapical tooth; tergite VI posterior margin without narrow teeth (Fig. 15F); longest setae on cerci distal segments greater than corresponding segment	***D. tabang* sp. nov.**
–	Tarsal claw with 2 developed subapical teeth; tergite VI posterior margin with narrow teeth (Fig. 15C, E); longest setae on cerci distal segments not greater than corresponding segment	**6**
6	Mesothorax marginal setae simple; forefemur transverse setae apices pointed (Fig. 14A); cerci brownish band present every four segments (Fig. 2A)	***D. bauernfeindi* sp. nov.**
–	Mesothorax marginal setae forked; forefemur transverse setae apices combed; cerci brownish band absent (Fig. 7A)	***D. vonrinteleni* sp. nov.**

## Discussion

The use of COI sequence as initial clustering method greatly facilitated the taxonomic pipeline and species discovery of the Philippine *Dudgeodes* representatives. The initial clustering allowed for more systematic analysis of nymph morphological characters, permitting the efficient production of species diagnoses and descriptions using more meaningful characters. Moreover, the initial clustering allowed a closer look on certain characters which have intraspecific variability (Figs 9A–H, 10A–H, 11A–E, 13A–D).

The 79 COI sequences analyzed gave 14–15 distinct clusters and singletons from the Philippines, with GMYC providing the more conservative number. Based on our morphological assessment, the clusters obtained from the GMYC better corresponds to morphological species concepts compared to PTP and mPTP. Several studies on mayflies have depicted that the GMYC putative species corresponds to biological species (Gattolliat and Monaghan 2010, Gattolliat et al. 2013, 2018, Vuataz et al. 2013, Hrivniak et al. 2019).

Luzon island harbors the highest diversity of *Dudgeodes* to date, with four species described (*D. pescadori*, *D. luntian* sp. nov., *D. vonrinteleni* sp. nov., and *D. freitagi* sp. nov.) and one undescribed cluster (PH5). This high diversity of a single genus in Luzon has been observed in the caddisfly genus *Hydropsyche* Pictet, 1834 and is partly attributed to the peculiar biogeographic history of the archipelago (Mey 2003). However, we also consider the sampling bias given that the university is on Luzon island and several course-projects (BIO-PHIL, https://bio-phil.net/) were done in Southern Luzon. It is likely that other islands (i.e., Palawan, Mindanao) would have higher number of *Dudgeodes* species too given the same sampling effort.

Most Philippine representatives including the undescribed clusters in this study are currently found in the respective island of the type locality only (*Dudgeodes vonrinteleni* sp. nov., *D. freitagi* sp. nov. and *D. pescadori* in Luzon, *D. bauernfeindi* sp. nov. in Negros, *D. tabang* sp. nov. in Leyte, PH4 and PH6 in Palawan) and are most likely island endemics. Although possibly due to missing localities included in this study, island endemics are quite common among aquatic insect species of the Philippines (Freitag and Balke 2011, Freitag 2013, Freitag and Zettel 2013, Komarek and Freitag 2014, Vidal et al. 2017, Garces et al. 2018).

Only two species are found on more than one island: *Dudgeodes luntian* sp. nov. in Luzon, Negros and Mindoro islands, *D. pangantihoni* sp. nov. in Mindanao and Camiguin islands. Haplogroups can be recognized in *D. luntian* sp. nov. that corresponds to the island distribution, and no haplotypes are shared between different islands (Fig. 1). However, this is not observed in *D. pangantihoni* sp. nov. as one haplotype is shared between greater Mindanao and Camiguin island suggesting either current or remnant intra-archipelagic dispersal crossing sea channels.

Interestingly, most major islands have more than one (putative) species (Fig. 1): Luzon with four species and one undescribed cluster, Palawan with two undescribed clusters (PH4, PH6), Negros with two species and one undescribed cluster (PH2), Leyte with one species and one underscribed singleton (PH1), and Mindanao with one species and one undescribed singleton (PH7). This further highlights the diversity not just of the entire archipelago but even within the major islands. The other clusters (fewer than five individuals) not described in detail here and samples representing the other intra-Philippine biogeographic regions (Biodiversity Laboratory, AdMU Project) will be the subject of a separate paper.

Given the current diversity in the Philippines and the Sunda islands (Sartori et al. 2008), it is expected that high number of still unknown species is awaiting discovery in continental Southeast Asia.

## Supplementary Material

XML Treatment for Dudgeodes bauernfeindi

XML Treatment for Dudgeodes freitagi

XML Treatment for Dudgeodes luntian

XML Treatment for Dudgeodes pangantihoni

XML Treatment for Dudgeodes tabang

XML Treatment for Dudgeodes vonrinteleni

XML Treatment for Dudgeodes celebensis

XML Treatment for Dudgeodes hutanis

XML Treatment for Dudgeodes pescadori

XML Treatment for Dudgeodes stephani

XML Treatment for Dudgeodes ulmeri
